# Multi-omics profiling identifies intestinal microbial shifts and tissue-specific immunometabolic changes associated with experimental *Nocardia seriolae* challenge in hybrid sturgeon (*Acipenser baerii*♀ × *Acipenser schrenckii*♂)

**DOI:** 10.3389/fmicb.2026.1956937

**Published:** 2026-09-18

**Authors:** Luyun Ni, Bo Xian, Feiyang Li, Qiaolin Zou, Jun Du, Jiansheng Lai, Ya Liu

**Affiliations:** Fisheries Research Institute, Sichuan Academy of Agricultural Sciences (Sichuan Fisheries Research Institute), Chengdu, China

**Keywords:** hybrid sturgeon, immunometabolism, intestinal microbiota, metabolome, *Nocardia seriolae*, transcriptome

## Abstract

**Introduction:**

*Nocardia seriolae* is an important bacterial pathogen in aquaculture, but the intestinal microbial, transcriptional, and metabolic responses of hybrid sturgeon (*Acipenser baerii*♀ × *Acipenser schrenckii*♂) to infection remain poorly understood.

**Methods:**

Hybrid sturgeons were experimentally challenged with N. seriolae. PBS-injected control fish were sampled at 0 days post-infection (dpi) as the baseline reference, whereas infected fish were sampled at 7 and 14 dpi for microbiome, transcriptome, and metabolome profiling. Full-length *16S rRNA* gene and ITS sequencing were used to characterize intestinal microbial communities, while RNA-seq and untargeted LC–MS metabolomics were performed on liver and spiral valve intestine tissues.

**Results:**

Compared with the baseline control group, fish sampled at 7 and 14 dpi showed differences in intestinal bacterial community composition, whereas bacterial alpha diversity did not differ significantly among the three sampled groups. ITS profiling also showed differences in fungal diversity and community composition among groups, although no fungal genus remained significant after false discovery rate (FDR) correction. RNA-seq revealed tissue-specific transcriptional responses, with the liver showing a larger number of differentially expressed genes than the spiral valve intestine. Hepatic pathways were mainly related to immune recognition, complement and coagulation cascades, antigen processing, and stress-related responses, whereas intestinal transcriptional changes involved phagosome, cell adhesion, and mucosal immune-related pathways. Metabolomic analysis showed tissue- and stage-related metabolic changes, with clearer pathway-level evidence in the spiral valve intestine at 14 dpi, particularly involving amino acid metabolism and protein digestion and absorption. Representative metabolites related to lipid and bile acid metabolism, carnitine-associated lipid utilization, energy metabolism, and purine/nicotinamide metabolism also showed sampling-stage-related variation. Genus–metabolite association analysis further identified candidate relationships between intestinal bacterial genera and representative metabolites.

**Discussion:**

These findings provide a multi-omics view of intestinal microbial and tissue-specific immunometabolic changes associated with experimental N. seriolae challenge in hybrid sturgeon. The candidate genus–metabolite relationships provide a basis for future targeted validation of microbial and metabolic features associated with nocardial infection.

## 1 Introduction

Sturgeon is one of the most valuable aquaculture fish groups because of its high-quality meat and caviar production. In China, hybrid sturgeon, including *Acipenser baerii*♀ × *Acipenser schrenckii*♂, has become an important cultured type owing to its growth performance and farming value. With the expansion of intensive culture, however, bacterial diseases have become a persistent problem in sturgeon farming. Several bacterial pathogens, such as *Aeromonas hydrophila* (Jiang et al., 2018; Luo et al., 2018), *Yersinia ruckeri* (Li et al., 2017), *Citrobacter freundii* (Yang J. et al., 2021), *Staphylococcus sciuri* (Zhang et al., 2022), and *Nocardia seriolae* (Ni et al., 2024), have been reported to cause disease or immune disturbance in sturgeons and other cultured fish species. These infections often involve tissue injury, intestinal dysfunction, and marked changes in host immune responses. For a species group with relatively limited genomic and immunological resources, understanding how sturgeon respond to bacterial infection remains a practical and biological question.

*N. seriolae* is the causative agent of fish nocardiosis and has been recognized as an important pathogen in both marine and freshwater aquaculture (Liu et al., 2023; Nawaz et al., 2022). Nocardiosis usually develops as a chronic and systemic disease, with granulomatous lesions frequently observed in immune and metabolism-related organs such as the spleen, kidney, and liver. Recent studies in fish have shown that *N. seriolae* infection can induce immune-related transcriptional changes, including pathways linked to pattern recognition, phagocytosis, antigen processing, cytokine signaling, cell death, and metabolic regulation (Chen J. et al., 2018; Dong et al., 2024). Our previous study also documented natural infection of hybrid sturgeon with *N. seriolae* and white sturgeon iridovirus, revealing severe pathological lesions and spleen transcriptomic changes related to host immune responses (Ni et al., 2024). Since that case involved natural infection and viral co-detection, the host response of hybrid sturgeon under experimental *N. seriolae* challenge still needs to be examined in a more controlled setting.

The intestine and liver are two key sites for understanding host responses to bacterial infection. The fish intestine is not only responsible for digestion and nutrient absorption, but also serves as an important mucosal immune surface where host cells, resident microorganisms, and dietary or microbial metabolites interact. In sturgeons, the spiral valve intestine is a distinctive intestinal structure and is closely associated with nutrient handling and local barrier function. Previous studies have shown that intestinal disorders in hybrid sturgeon are often accompanied by shifts in microbial composition, including changes in dominant genera such as *Clostridium*, *Cetobacterium*, *Mycoplasma*, *Candidatus Arthromitus*, and opportunistic bacterial taxa (Huang et al., 2020; Peng et al., 2026). The liver is a central organ for metabolic and immune regulation, contributing to acute-phase responses, complement activity, detoxification, lipid metabolism, and redox homeostasis. Transcriptomic studies in sturgeons challenged with bacterial pathogens have repeatedly highlighted immune pathways such as Toll-like receptor signaling, NOD-like receptor signaling, complement and coagulation cascades, phagocytosis, and antigen processing and presentation (Costábile et al., 2022; Jiang et al., 2018; Li et al., 2017; Luo et al., 2018).

High-throughput omics approaches have made it possible to observe infection responses from several connected layers. Microbiome profiling describes community-level changes in intestinal microorganisms; RNA-seq captures host transcriptional responses; metabolomics reflects biochemical changes closer to physiological phenotype. In fish bacterial infection studies, these approaches have been used to reveal changes in intestinal microbiota, immune gene expression, amino acid metabolism, lipid metabolism, energy metabolism, and host-microbe associations (Chen et al., 2025; Dong et al., 2024; Zhang et al., 2023). In sturgeons, however, most available infection-related omics studies have focused on the spleen, head kidney, or intestinal microbiota alone, and only a few have combined gut microbial profiling with metabolic or transcriptomic data (Jiang et al., 2018; Luo et al., 2018; Ni et al., 2024; Yang J. et al., 2021). Thus, a parallel analysis of intestinal microbial profiles, intestinal and hepatic transcriptomes, and tissue metabolomes may provide a broader view of how hybrid sturgeon respond to *N. seriolae* infection.

In the present study, hybrid sturgeons were experimentally challenged with *N. seriolae*. PBS-injected control fish were sampled at 0 dpi as the baseline reference, whereas infected fish were sampled at 7 and 14 dpi. Full-length 16S rRNA and ITS sequencing were used to characterize bacterial and fungal communities in intestinal contents. RNA-seq and untargeted LC–MS metabolomics were performed on liver and spiral valve intestine tissues. In addition, pathway-level cross-omics comparison and genus–metabolite association analyses were conducted to examine the microbiome, transcriptome, and metabolome patterns across the three sampled groups. This study aimed to describe intestinal microbial and tissue-specific immunometabolic changes associated with experimental *N. seriolae* challenge in hybrid sturgeon.

## 2 Materials and methods

### 2.1 Ethics statement

All procedures involving live fish were conducted in accordance with the institutional guidelines for the care and use of experimental animals in China and were approved by the Animal Ethics Committee of the Fisheries Research Institute, Sichuan Academy of Agricultural Sciences (Sichuan Fisheries Research Institute; Approval No. 20250224003A). During acclimation and experimental infection, fish were maintained under controlled aquaculture conditions, and all handling procedures were performed to minimize unnecessary stress and suffering. Prior to necropsy and tissue collection, fish were deeply anesthetized and euthanized by immersion in 200 mg/L MS-222.

### 2.2 Bacterial strain, experimental fish and challenge design

The *N. seriolae* strain HS231120 used in this study was originally isolated from a natural disease outbreak in cultured hybrid sturgeon (*A. baerii*♀ × *A. schrenckii*♂) in Sichuan Province, China. Before the challenge experiment, the cryopreserved isolate was recovered from glycerol stock and purified on brain heart infusion agar (BHI; HuanKai Microbial, Guangdong, China) at 28 °C. For preparation of the challenge inoculum, a single colony was transferred into BHI broth and incubated at 28 °C with shaking at 180 rpm for 3 d. Bacterial cells were then collected by centrifugation, washed twice with sterile phosphate-buffered saline (PBS), and resuspended in sterile PBS. The bacterial suspension was adjusted to 1 × 10^7^ CFU/mL, and the final concentration was verified by serial dilution and plate counting on BHI agar.

Healthy hybrid sturgeons (*A. baerii*♀ × *A. schrenckii*♂) were obtained from a commercial farm in Sichuan Province, China, and acclimated for 1 week in a recirculating aquaculture system maintained at 22 °C before experimental infection. The fish had an average body weight of 60.88 ± 4.27 g. Before challenge, randomly selected fish were screened by PCR targeting the *16S rRNA* gene and heat shock protein 65 (*hsp65*) of *N. seriolae* to confirm the absence of the pathogen. Fish were randomly assigned to three sampling groups: a PBS-injected baseline control group sampled at 0 days post-infection (0 dpi), and two *N. seriolae*-infected groups sampled at 7 and 14 dpi, respectively. Each group consisted of four independent replicate tanks, with 15 fish maintained in each tank. Fish in the infected groups were intraperitoneally injected with 200 μL of freshly prepared *N. seriolae* HS231120 suspension at a concentration of 1 × 10^7^ CFU/mL, whereas fish in the control group received an equal volume of sterile PBS. The control fish were sampled once and used as the baseline reference for comparisons with the infected groups. After injection, all fish were maintained under the same environmental and husbandry conditions at 22 °C and monitored daily for clinical signs, behavioral changes, and mortality until sampling.

### 2.3 Confirmation of experimental infection

To confirm experimental infection, liver, and spleen were aseptically collected from infected fish at 7 and 14 dpi. Samples from the PBS-injected control fish were collected at 0 dpi and analyzed as the baseline reference. Bacterial isolation was performed by streaking tissue samples onto brain heart infusion agar, followed by incubation at 28 °C. Suspected *Nocardia* colonies were purified and subjected to molecular identification based on partial *16S rRNA* and *hsp65* gene sequences. Genomic DNA was extracted from purified bacterial isolates using a commercial bacterial DNA extraction kit according to the manufacturer’s instructions. The *16S rRNA* gene was amplified using the primers F (5′-AGA GTT TGA TCC TGG CTC AG-3′) and R (5′-GGC TAC CTT GTT ACG ACT T-3′). A partial fragment of the hsp65 gene was amplified using the primers F (5′-ACC AAC GAT GGT GTG TCC AT-3′) and R (5′-CTT GTC GAA CCG CAT ACC CT-3′) (Lei et al., 2020). The expected amplicon sizes were approximately 1,500 bp for the *16S rRNA* gene and 400 bp for the *hsp65* gene. PCR products were examined by agarose gel electrophoresis and sequenced. The resulting sequences were compared with reference sequences in GenBank using BLAST to confirm the identity of the isolates as *N. seriolae*. Baseline samples from the PBS-injected control group were analyzed using the same procedures.

### 2.4 Sample collection and multi-omics experimental overview

Based on the pronounced hepatic lesions and spiral valve intestinal alterations observed in our previous pathological assessment of *N. seriolae* infection, the liver and spiral valve intestine were selected as representative tissues for evaluating systemic and local host changes, respectively. Samples for multi-omics profiling were collected from the PBS-injected baseline control group at 0 dpi and from infected fish at 7 and 14 dpi. Before sampling, fish were euthanized as described above, and necropsy was performed under aseptic conditions. For each group, 4 fish were selected, with one fish obtained from each of the four replicate tanks. Intestinal contents from these fish were used for full-length 16S rRNA and ITS sequencing, while liver and spiral valve intestine tissues from the same fish were used for RNA-seq. Thus, the 4 biological replicates used for microbiome and RNA-seq analyses represented 4 separate tanks. For untargeted metabolomics, the same 4 fish were analyzed together with 2 additional fish selected from 2 different tanks, resulting in a tank allocation of 1, 1, 2, and 2 fish per group. All tissue samples were rinsed briefly with sterile PBS when necessary, immediately frozen in liquid nitrogen, and stored at −80 °C until analysis. The overall experimental design is shown in Figure 1, and the detailed sample allocation and cross-omics correspondence are provided in Supplementary Table S1.

**Figure 1:**
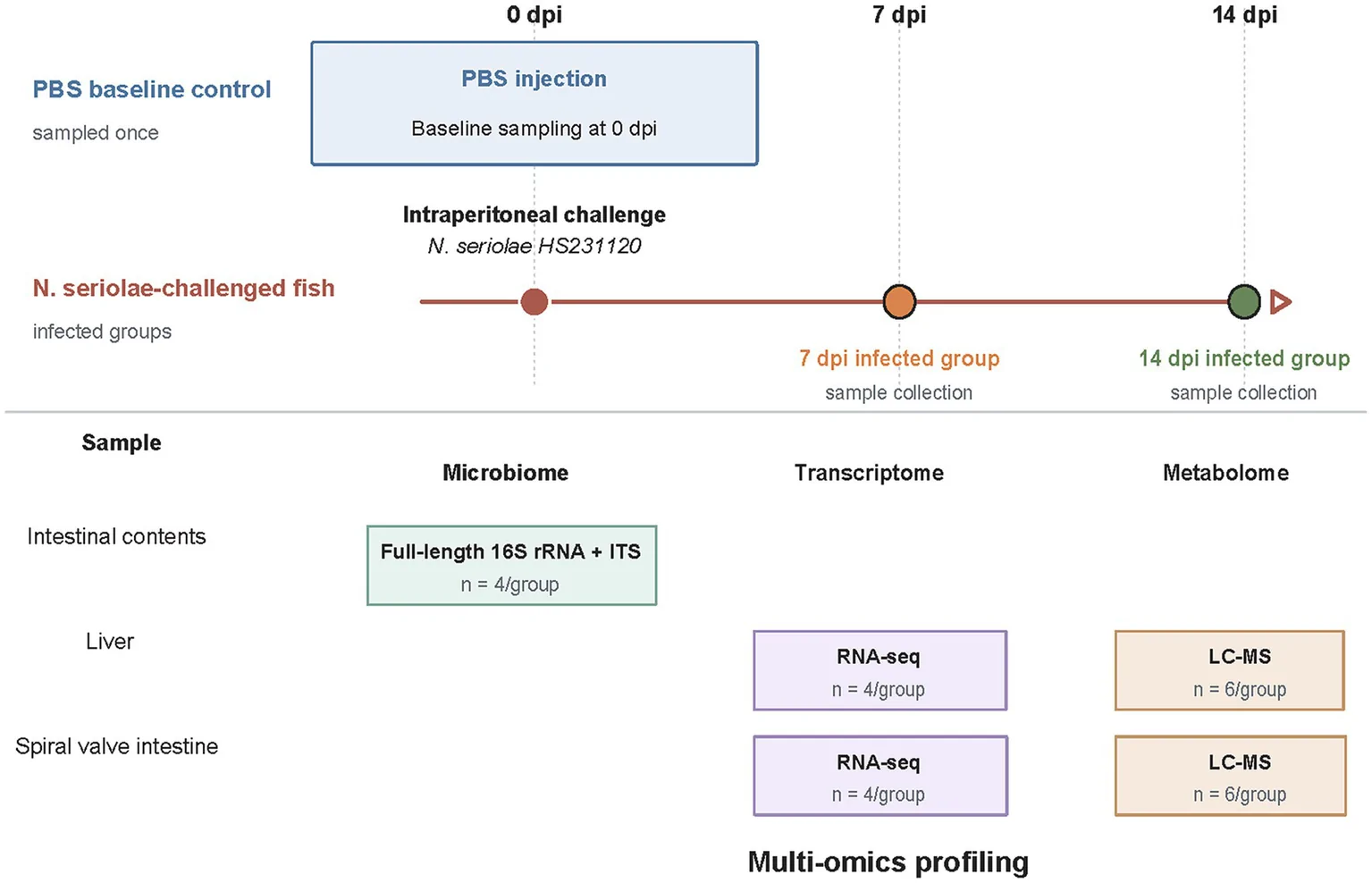
Multi-omics experimental design. Hybrid sturgeons were assigned to PBS-injected baseline control group sampled at 0 dpi and *Nocardia seriolae*-infected groups sampled at 7 and 14 dpi. Intestinal contents were used for 16S/ITS profiling, while liver and spiral valve intestine tissues were used for RNA-seq and LC–MS metabolomics.

### 2.5 Full-length 16S rRNA and ITS amplicon sequencing

Microbial DNA was extracted from intestinal content samples using the E.Z.N.A. Soil DNA Kit (Omega Bio-tek, Norcross, GA, USA) following the manufacturer’s instructions. The concentration and integrity of the extracted DNA were evaluated by agarose gel electrophoresis and spectrophotometric analysis before PCR amplification. For bacterial community profiling, the nearly full-length 16S rRNA gene covering the V1–V9 region was amplified using the primer pair 27F (5′-AGRGTTYGATYMTGGCTCAG-3′) and 1492R (5′-RGYTACCTTGTTACGACTT-3′). For fungal community profiling, the ITS region was amplified using ITS1F (5′-CTTGGTCATTTAGAGGAAGTAA-3′) and ITS4R (5′-TCCTCCGCTTATTGATATGC-3′). PCR amplification was performed in triplicate for each sample to reduce amplification bias, and the resulting amplicons were checked by agarose gel electrophoresis. PCR products with the expected fragment size were purified using the AxyPrep DNA Gel Extraction Kit (Axygen Biosciences, Union City, CA, USA) and quantified using Qubit fluorometric quantification. Purified amplicons from different samples were pooled in appropriate proportions according to their concentrations. SMRTbell libraries were then constructed from the pooled barcoded amplicons using a PacBio library preparation kit following the manufacturer’s protocol. Sequencing was performed on the PacBio Sequel IIe platform by Shanghai Biozeron Biotechnology Co., Ltd. (Shanghai, China).

### 2.6 Microbiome data processing and ecological statistics

Raw PacBio sequencing reads were processed using SMRT Link to generate circular consensus sequence (CCS) reads. CCS reads were demultiplexed according to barcode sequences, and primer and barcode sequences were removed before downstream analysis. Low-quality reads and sequences outside the expected amplicon length range were discarded. For bacterial 16S rRNA data, high-quality sequences were clustered into operational taxonomic units (OTUs) at 98.65% sequence similarity using UPARSE v10 (Edgar, 2013), with potential chimeric sequences removed during clustering. Representative OTU sequences were taxonomically assigned against the SILVA SSU r138.1 database (Quast et al., 2013) using a confidence threshold of 80%. Fungal ITS sequences were clustered into OTUs at 98.65% similarity using USEARCH v10, and representative sequences were annotated using BLAST+ v2.12.0 against the NT-fungi database (v202307; E-value ≤ 1 × 10^−20^).

Bacterial alpha diversity was evaluated using observed species, Faith’s phylogenetic diversity, Shannon index, and Simpson index, together with additional richness and evenness indices where applicable. Differences in alpha-diversity indices among the control, 7-dpi, and 14-dpi groups were evaluated using non-parametric statistical tests. Beta diversity was calculated based on Bray-Curtis, weighted UniFrac, and unweighted UniFrac distance matrices and visualized by principal coordinate analysis (PCoA). PERMANOVA was used to test differences in bacterial community composition among groups, and PERMDISP was performed to evaluate whether the observed group separation was associated with differences in within-group dispersion. Additional UniFrac-based beta-diversity metrics were evaluated when phylogenetic distance information was available. The relative abundance of dominant bacterial phyla was summarized to characterize community patterns across the sampled groups. Before genus-level compositional analysis, genera detected in fewer than 3 of the 12 samples or with a mean relative abundance below 0.01% were removed. A pseudocount of 0.5 was added to the count data, followed by centered log-ratio transformation. Pairwise comparisons were performed using exact permutation tests for 7 dpi versus control, 14 dpi versus control, and 14 dpi versus 7 dpi. Mean CLR differences and bootstrap 95% confidence intervals were calculated, and *p* values were adjusted using the Benjamini–Hochberg method.

Fungal alpha diversity was evaluated using observed species, Faith’s phylogenetic diversity, Shannon index, and Simpson index, with overall group differences assessed using the Kruskal–Wallis test. Bray–Curtis PCoA, PERMANOVA, and PERMDISP with 9,999 permutations were used to evaluate fungal community composition. Genus-level fungal data were analyzed using the same filtering, CLR transformation, exact permutation, bootstrap confidence interval, and FDR-correction procedures described above for the bacterial data.

### 2.7 RNA extraction, library construction and transcriptome sequencing

Total RNA was extracted from liver and spiral valve intestine samples using TRIzol reagent (Invitrogen, Carlsbad, CA, USA) according to the manufacturer’s instructions. RNA concentration, purity, and integrity were assessed using an Agilent 2,100 Bioanalyzer (Agilent Technologies, Palo Alto, CA, USA) and RNase-free agarose gel electrophoresis. Only RNA samples with acceptable integrity and quality were used for library preparation. For each sample, eukaryotic mRNA was enriched using oligo(dT) magnetic beads and fragmented into short fragments in fragmentation buffer. First-strand cDNA was synthesized using random primers, followed by second-strand cDNA synthesis with DNA polymerase I, RNase H, dNTPs, and reaction buffer. The resulting cDNA fragments were purified, end-repaired, adenylated at the 3′ ends, and ligated to Illumina sequencing adapters. After size selection and PCR amplification, the libraries were sequenced on the Illumina HiSeq 4,000 platform by Gene Denovo Biotechnology Co., Ltd. (Guangzhou, China).

### 2.8 RNA-seq data processing and functional enrichment analysis

A full-length reference transcriptome was first constructed by pooling liver and spiral valve intestine RNA from the baseline control (0 dpi) and the 7- and 14-dpi challenged groups. The pooled RNA was sequenced on the PacBio Revio platform, processed using Iso-Seq v4.3.0, and dereplicated using CD-HIT v4.8.1 at 90% sequence identity. Raw RNA-seq reads were filtered using fastp v0.18.0 (Chen S. et al., 2018) to remove adapter-contaminated reads, reads containing more than 10% unknown nucleotides, and low-quality reads with more than 50% bases having a quality score of Q ≤ 20. The clean reads from individual samples were mapped to this isoform reference, and gene abundance and expected counts were estimated using RSEM v1.2.19 with default parameters (Li and Dewey, 2011). RPKM values were calculated for expression-level visualization and sample-level quality assessment, including principal component analysis (PCA) and Pearson correlation analysis. Differential expression analysis was performed using DESeq2 v1.20.0 (Love et al., 2014) based on gene-level count data. Pairwise comparisons were conducted as 7 dpi vs. control, 14 dpi vs. control, and 14 dpi vs. 7 dpi for both liver and spiral valve intestine samples. Genes with a false discovery rate (FDR) < 0.05 and an absolute |log2(fold change)| ≥ 1 were defined as differentially expressed genes (DEGs). In each comparison, upregulated and downregulated genes were defined relative to the first group in the comparison; for example, “7 dpi vs control” indicates genes upregulated or downregulated in the 7-dpi group relative to the control group. Functional enrichment analysis of DEGs was performed using GO.db v3.14.0 and KEGG Release 101. GO terms and KEGG pathways significantly enriched among DEGs were identified using hypergeometric tests followed by FDR correction, with FDR ≤ 0.05 considered statistically significant.

### 2.9 qRT-PCR validation of RNA-seq results

To validate the RNA-seq results, 10 DEGs were selected for quantitative real-time PCR (qRT-PCR) analysis, including *CFB*, *MIF*, *HLA-DPB1*, *CTSL* and *LPL* from the liver, and *CLEC17A*, *H2-Aa*, *MYD88*, *NOS2* and *FTH1* from the spiral valve intestine. These genes were chosen to cover major functional categories observed in the transcriptomic analysis. qRT-PCR was performed using gene-specific primers, and the primers used for target and candidate reference genes are listed in Supplementary Table S2. Candidate reference genes, including *β-actin*, glyceraldehyde-3-phosphate dehydrogenase (*GAPDH*) and eukaryotic translation elongation factor 1 alpha (*EF1α*), were evaluated for expression stability before normalization using NormFinder. Relative expression levels were calculated using the 2^−ΔΔCt^ method, with the control group used as the calibrator. Each assay was conducted in triplicate for each sample. The agreement between RNA-seq and qRT-PCR results was assessed using log_2_ fold-change values at 7 and 14 dpi relative to the control group.

### 2.10 Untargeted metabolomics profiling and data processing

Untargeted metabolomic profiling was performed on liver and spiral valve intestine samples. Approximately 100 mg of each tissue sample was mixed with 1 mL of pre-cooled methanol/acetonitrile/water extraction solution and homogenized under low-temperature conditions. The homogenates were subjected to ultrasonic extraction, incubated at −20 °C to promote protein precipitation, and centrifuged at 13,000–14,000 × g at 4 °C. The supernatants were collected and vacuum-dried. Before LC–MS/MS analysis, dried extracts were reconstituted in acetonitrile/water solution, vortexed thoroughly, and centrifuged again to obtain clarified supernatants for injection. Quality control (QC) samples were prepared by pooling equal aliquots from all biological samples and were inserted throughout the analytical sequence to monitor instrumental stability and data reproducibility. Metabolomic data were acquired in both positive and negative ion modes using an ultra-high-performance liquid chromatography system coupled to a high-resolution mass spectrometer at Gene Denovo Biotechnology Co., Ltd. (Guangzhou, China). Chromatographic separation was performed on a HILIC column, and samples were analyzed in a randomized injection order to reduce systematic bias. Raw mass spectrometry data were converted to mzML format using ProteoWizard (Chambers et al., 2012). Peak detection, retention-time correction, peak alignment, and peak-area extraction were performed using XCMS (Smith et al., 2006). Features with excessive missing values were removed, missing values were imputed, and features with poor reproducibility in QC samples were filtered before downstream statistical analysis. Metabolite annotation was performed by matching accurate mass information, retention time information where available, and MS/MS spectra against an in-house reference database. Unless otherwise confirmed by authentic standards, metabolite identities were considered putative annotations based on spectral matching, broadly corresponding to MSI level 2.

### 2.11 Differential metabolite and pathway enrichment analysis

After data preprocessing and metabolite annotation, the positive- and negative-ion datasets were integrated for downstream statistical analysis. PCA and sample correlation analysis were used to evaluate overall data quality, sample distribution, and the clustering of pooled QC samples. Differential metabolites were identified for pairwise comparisons among the control, 7-dpi, and 14-dpi groups in liver and spiral valve intestine samples. Orthogonal partial least-squares discriminant analysis (OPLS-DA) was performed using the ropls package in R (Thévenot et al., 2015), and the reliability of the model was evaluated by cross-validation and permutation testing where applicable. Metabolites with variable importance in projection (VIP) ≥ 1 and *p* < 0.05 were considered differentially abundant metabolites, and FDR-adjusted *p* values were reported to indicate the robustness of individual metabolite changes after multiple-testing correction. Differential metabolites were annotated to KEGG pathways for pathway enrichment analysis. Enrichment significance was evaluated using hypergeometric tests followed by FDR correction. Pathways with FDR < 0.05 were considered statistically significant, whereas pathways with *p* < 0.05 but FDR ≥ 0.05 were interpreted as nominal enrichment signals. The primary metabolomic analysis used individual-fish profiles. To assess potential within-tank dependence, normalized log₂-transformed abundances from fish within the same tank were averaged, yielding four tank-level profiles per group. Agreement between the individual-fish and tank-level analyses was evaluated using Spearman correlations of log₂ fold changes and the directional concordance of differential metabolites.

### 2.12 Cross-omics comparison and statistical analysis

To compare the transcriptomic and metabolomic results, KEGG pathways identified in the two datasets were matched by pathway ID for each tissue and comparison. The analysis summarized the numbers of shared pathway annotations, RNA-seq pathways with FDR < 0.05, metabolomic pathways with FDR < 0.05, and pathways that reached FDR < 0.05 in both datasets. Nine-quadrant plots were further used to compare the log₂FC directions of candidate gene–metabolite pairs in the liver and spiral valve intestine. Sample-level gene–metabolite associations were examined using normalized gene-expression and metabolite-abundance data from 12 matched samples for each tissue. The initial candidates were restricted to pairs with |Spearman ρ| ≥ 0.95 and BH-adjusted *p* < 0.05. The genes and metabolites were also required to meet their respective differential-feature criteria in at least one comparison. Sampling-group effects were then adjusted using partial Spearman correlation. Gene–metabolite pairs sharing at least one KEGG pathway annotation and meeting partial *p* < 0.05 and BH-adjusted partial *p* < 0.10 were retained for network visualization. These associations were considered exploratory. Exploratory bacterial genus–metabolite associations were analyzed using 12 matched intestinal samples, with one fish from each replicate tank included in each group. The genus pool was restricted to the 20 genera with the highest mean relative abundance, and representative metabolites were selected from the differential metabolite results. Spearman correlations were calculated between genus relative abundances and log-transformed metabolite abundances. Significance was assessed using 4,999 permutations followed by Benjamini–Hochberg correction. Only associations with BH-adjusted *p* < 0.10 were retained for visualization. Because no bacterial genus reached FDR < 0.05 in the CLR-based differential-abundance analysis, these associations were treated as exploratory. The ITS dataset was incorporated into the multi-omics interpretation at the community level. Because ITS amplicon sequencing does not directly establish fungal metabolic functions, fungal taxa were not assigned to KEGG pathways. A *post hoc* fungal genus–metabolite association network was not constructed, and the fungal results were interpreted primarily on the basis of alpha diversity, community composition, and genus-level compositional patterns.

Statistical analyses were performed in R v4.3.3. For comparisons among three groups, non-parametric tests or one-way analysis of variance followed by *post hoc* tests were used depending on data distribution and homogeneity of variance. For omics-wide analyses, multiple-testing correction was performed using the Benjamini-Hochberg FDR method. *p* < 0.05 was considered statistically significant; unless otherwise specified, FDR < 0.05 was considered statistically significant after multiple-testing correction. Graphs were generated using R packages including ggplot2 and pheatmap where applicable.

## 3 Results

### 3.1 Multi-omics experimental design and dataset overview

Following experimental challenge, infected fish exhibited lethargic swimming and body curvature. Gross examination revealed numerous small white nodules on the liver surface. No obvious abnormalities were observed in the baseline control fish sampled at 0 dpi. Suspected *Nocardia* colonies were re-isolated from the liver of challenged fish at 7 and 14 dpi. Partial *16S rRNA* and *hsp65* gene sequences of the recovered isolates showed high sequence similarity to reference sequences of *N. seriolae* in GenBank, confirming successful experimental infection. No *N. seriolae* was isolated or detected from the control fish. A multi-omics profiling strategy was applied to intestinal contents, liver, and spiral valve intestine samples collected from the control group and from infected fish at 7 and 14 dpi (Figure 1). The dataset included full-length 16S rRNA and ITS amplicon sequencing of intestinal contents, RNA-seq profiling of liver and spiral valve intestine, and untargeted LC–MS metabolomics of the same two tissues. This design enabled parallel assessment of intestinal microbial community profiles, tissue-specific transcriptional responses, metabolic changes, and cross-omics associations following *N. seriolae* infection.

### 3.2 Intestinal microbiome diversity and taxonomic composition

Bacterial alpha diversity in intestinal contents was compared among the baseline control (0 dpi), 7-dpi, and 14-dpi groups. Faith’s phylogenetic diversity, observed species, Shannon index, and Simpson index showed no significant differences among groups (Figure 2A). The additional alpha-diversity indices Chao1, ACE, and Pielou’s evenness showed the same pattern, with all pairwise *p* values greater than 0.05 (Table 1). Bacterial beta diversity was evaluated using Bray-Curtis, unweighted UniFrac, and weighted UniFrac distances. In the Bray-Curtis PCoA, samples from the three groups showed partial separation (Figure 2B). PERMANOVA based on Bray-Curtis distance detected a significant group effect (*R^2^* = 0.288, *p* = 0.024), while PERMDISP was not significant (*p* = 0.220; Table 2). Unweighted UniFrac also showed a significant group effect in PERMANOVA (*R^2^* = 0.216, *p* = 0.007), whereas weighted UniFrac did not reach significance (*p* = 0.160). PERMDISP tests based on unweighted and weighted UniFrac distances were also not significant (*p* = 0.107 and *p* = 0.485, respectively). At the phylum level, the bacterial community was mainly composed of Firmicutes, Proteobacteria, Bacteroidetes, Actinobacteria, Cyanobacteria, Fusobacteria, Chloroflexi, Verrucomicrobia, Deinococcus-Thermus, and Acidobacteria (Figure 2C). The relative abundances of these phyla varied across the three groups. Firmicutes accounted for a larger proportion at 7 dpi, while Proteobacteria showed a lower proportion at 7 and 14 dpi than in the baseline control group. Genus-level CLR-based compositional analysis identified several candidate changes across the three comparisons; however, no bacterial genus remained significant after FDR correction (Figure 2D). These results were therefore interpreted as compositional trends rather than statistically supported differential genera. ITS profiling revealed group-associated variation in the intestinal fungal community (Figure 3). Observed species richness, Shannon diversity, and Simpson diversity showed significant overall differences among the three sampled groups (Kruskal–Wallis *p* = 0.044, 0.037, and 0.017, respectively), whereas the difference in Faith’s phylogenetic diversity did not reach statistical significance (*p* = 0.069). Fungal community composition also differed among groups (PERMANOVA, *R^2^* = 0.483, *p* = 0.001), with no evidence of unequal multivariate dispersion (PERMDISP, *p* = 0.126). Fusarium, Aspergillus, Kazachstania, Cylindrium, Exophiala, Pichia, Geotrichum, Alternaria, and Millerozyma were among the dominant fungal genera, although their relative abundances varied among individual samples (Figure 3C; Supplementary Table S3). Despite several relatively large CLR effect estimates, no fungal genus remained significant after FDR correction.

**Figure 2:**
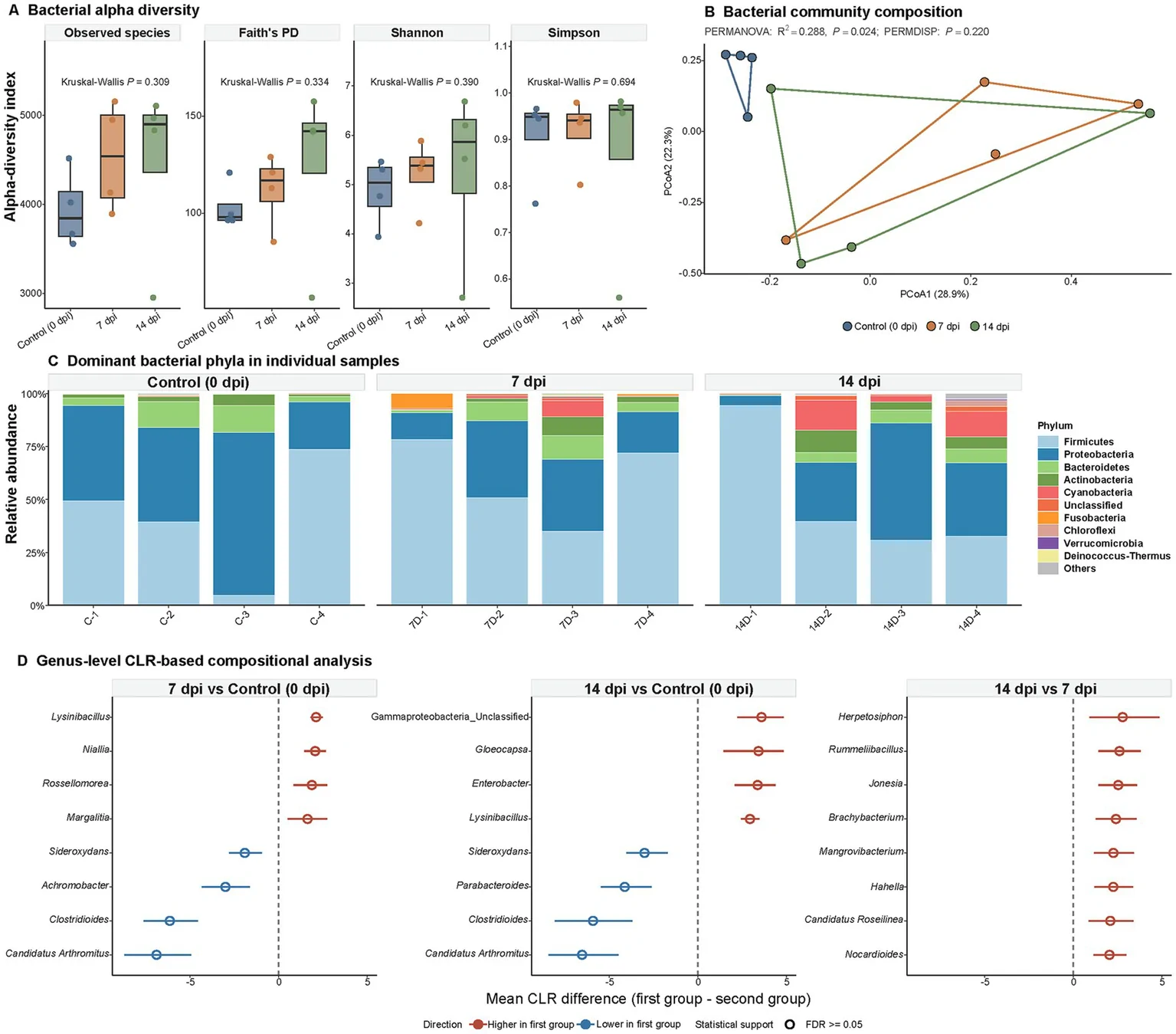
Intestinal bacterial diversity and taxonomic composition. **(A)** Alpha-diversity indices of bacterial communities in intestinal contents. **(B)** Bray–Curtis PCoA of bacterial community composition, with PERMANOVA and PERMDISP results shown in the panel. **(C)** Relative abundances of dominant bacterial phyla. **(D)** Genus-level CLR-based compositional analysis. Points indicate mean CLR differences between the first and second groups, and horizontal lines indicate bootstrap 95% confidence intervals. The eight genera with the lowest FDR values in each comparison are shown. No bacterial genus reached FDR < 0.05.

**Table 1:** Pairwise tests of bacterial alpha diversity indices.

Alpha index	7 dpi vs 14 dpi	Control vs 14 dpi	Control vs 7 dpi
Observed_species	0.990	0.585	0.510
Chao1	0.986	0.610	0.517
ACE	0.974	0.634	0.508
Shannon	0.997	0.876	0.909
Simpson	0.874	0.916	0.995
Pielou_J	0.998	0.915	0.938
Pd_faith	0.821	0.582	0.909

All *p*-values were greater than 0.05, indicating no significant pairwise differences in bacterial alpha-diversity indices among the sampled groups.

**Table 2:** Beta-diversity and multivariate-dispersion tests for the bacterial microbiome.

Distance	Test	*R* ^2^	*F*	Statistic	*p*-value
Bray_Curtis	PERMANOVA	0.288	1.824		0.023
Bray_Curtis	ANOSIM			0.340	0.024
Bray_Curtis	MRPP			0.072	0.806
Unweighted_Unifrac	PERMANOVA	0.216	1.237		0.007
Unweighted_Unifrac	ANOSIM			0.368	0.014
Unweighted_Unifrac	MRPP			0.021	0.833
Weighted_Unifrac	PERMANOVA	0.263	1.608		0.160
Weighted_Unifrac	ANOSIM			0.167	0.125
Weighted_Unifrac	MRPP			0.062	0.187
Bray_Curtis	PERMDISP		1.593		0.257
Unweighted_Unifrac	PERMDISP		2.444		0.107
Weighted_Unifrac	PERMDISP		0.793		0.485

PERMDISP was added to evaluate whether significant PERMANOVA results could be influenced by differences in within-group dispersion.

**Figure 3:**
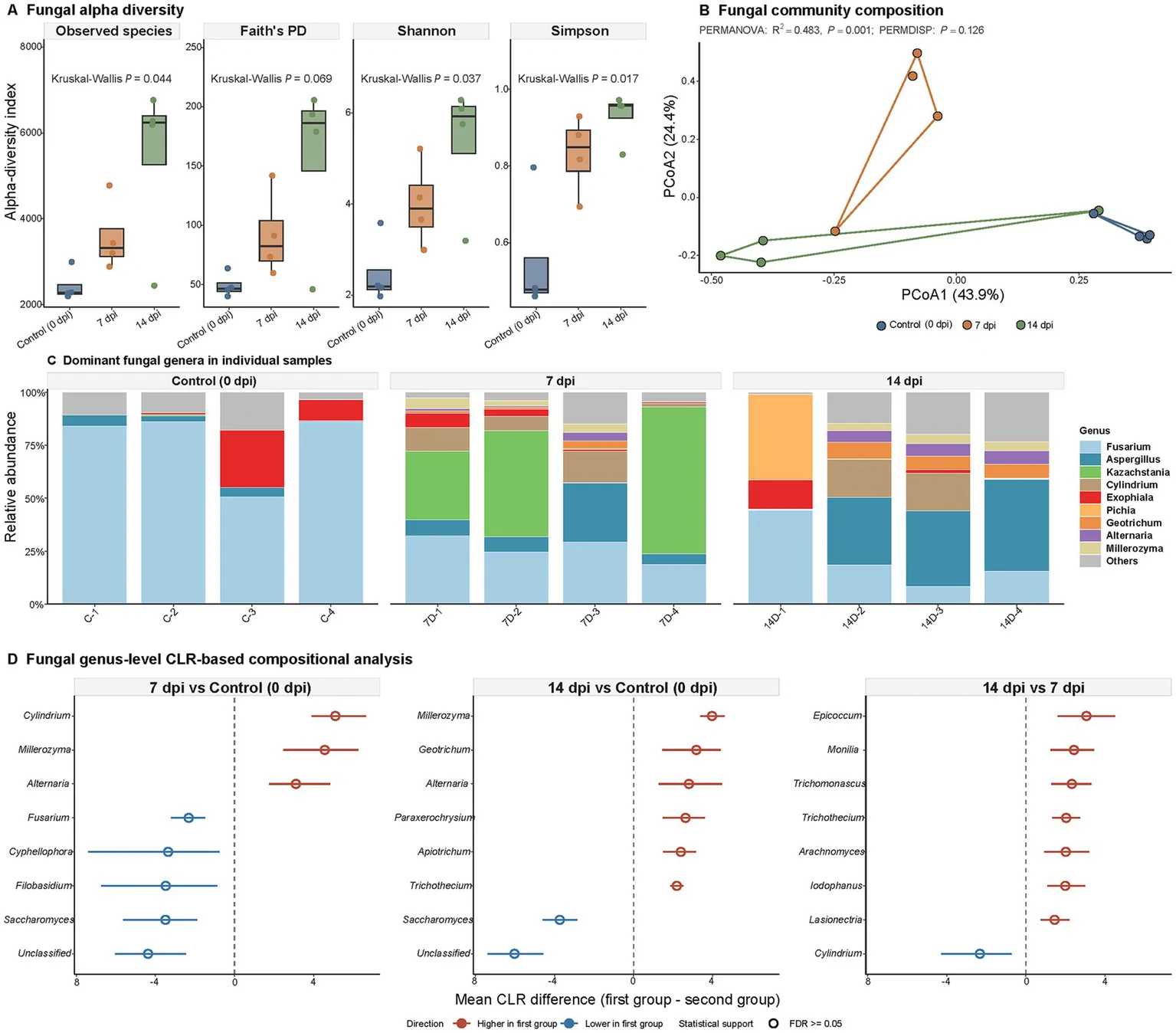
Intestinal fungal community profiles across the baseline control (0 dpi), 7-dpi, and 14-dpi groups. **(A)** Fungal alpha-diversity indices, including observed species, Faith’s PD, Shannon index, and Simpson index. Overall differences among groups were evaluated using the Kruskal–Wallis test. **(B)** PCoA of fungal community composition based on Bray–Curtis distances. PERMANOVA and PERMDISP were performed using 9,999 permutations. **(C)** Relative abundances of the dominant fungal genera in the 12 individual samples. **(D)** Genus-level CLR-based differential-abundance analysis. Points represent mean CLR differences between the first and second groups, and horizontal lines indicate bootstrap 95% confidence intervals. The eight genera with the lowest FDR values in each comparison are shown. *p*-values were adjusted using the Benjamini–Hochberg method, and no fungal genus remained significant at FDR < 0.05. Each group included four fish from four replicate tanks.

### 3.3 Transcriptomic profiles and KEGG enrichment in liver and spiral valve intestine

RNA-seq was performed to compare transcriptional profiles in the liver and spiral valve intestine across the baseline control (0 dpi), 7-dpi, and 14-dpi groups. PCA and sample correlation analysis showed clear tissue separation and overall consistency among RNA-seq samples (Supplementary Figure S1). Differential expression analysis showed that the number of DEGs varied between tissues and sampled time points (Figure 4A; Table 3). In the liver, 6,093 DEGs were identified in the 7 dpi compared with the control, including 4,584 upregulated and 1,509 downregulated genes. A similar number of DEGs was detected at 14 dpi, with 6,092 DEGs, including 4,454 upregulated and 1,638 downregulated genes. The comparison between 14 and 7 dpi identified 2,382 DEGs, including 1,340 upregulated and 1,042 downregulated genes. In the spiral valve intestine, 2,341 DEGs were detected at 7 dpi compared with the control group, including 1,647 upregulated and 694 downregulated genes. At 14 dpi, 1,510 DEGs were identified, with 717 upregulated and 793 downregulated genes. The comparison between 14 and 7 dpi identified 1,222 DEGs, including 329 upregulated and 893 downregulated genes.

**Figure 4:**
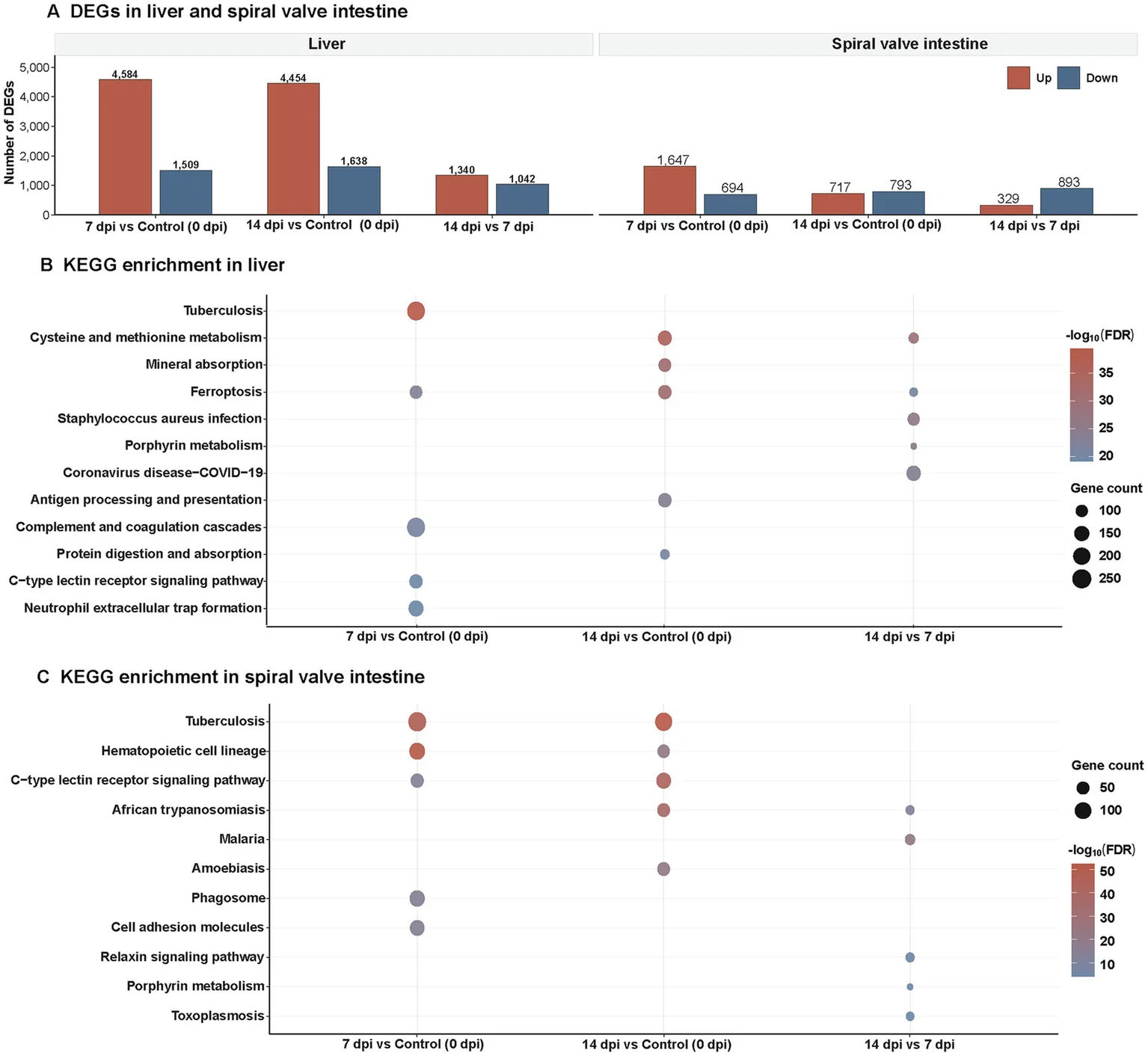
Transcriptomic profiles and KEGG enrichment in liver and spiral valve intestine. **(A)** Numbers of upregulated and downregulated differentially expressed genes (DEGs) in each comparison. Up and down indicate genes increased or decreased in the first group relative to the second group. **(B)** KEGG enrichment of DEGs in the liver. **(C)** KEGG enrichment of DEGs in the spiral valve intestine. Bubble size represents gene count, and color indicates −log_10_(*FDR*).

**Table 3:** Differentially expressed genes in liver and spiral valve intestine.

Tissue	Comparison	Upregulated	Downregulated	Total DEGs
Liver	7 dpi vs. Control	4,584	1,509	6,093
Liver	14 dpi vs. Control	4,454	1,638	6,092
Liver	14 dpi vs. 7 dpi	1,340	1,042	2,382
Spiral valve intestine	7 dpi vs. Control	1,647	694	2,341
Spiral valve intestine	14 dpi vs. Control	717	793	1,510
Spiral valve intestine	14 dpi vs. 7 dpi	329	893	1,222

Source labels containing Sp were relabeled as Intestine according to the experimental annotation.

KEGG enrichment analysis showed tissue- and stage-related enrichment patterns (Figures 4B,C; Table 4). In the liver, the main pathways enriched at 7 dpi included tuberculosis, ferroptosis, complement and coagulation cascades, C-type lectin receptor signaling, and neutrophil extracellular trap formation. Among the DEGs annotated to these pathways were *NOS2*, *FTH1*, *F13B*, *CD209*, and *NCF4*. At 14 dpi, the enriched pathways included cysteine and methionine metabolism, mineral absorption, ferroptosis, antigen processing and presentation, and protein digestion and absorption, involving DEGs such as *TAT*, *HMOX1*, *TF1*, *HSP90A.1*, and *COL3A1*. The comparison between 14 and 7 dpi highlighted cysteine and methionine metabolism, *Staphylococcus aureus* infection, porphyrin metabolism, and ferroptosis, which included *BHMT*, *H2-Aa*, *FTH*, *CFB*, and *TF2*. In the spiral valve intestine, the main pathways enriched at 7 dpi included hematopoietic cell lineage, tuberculosis, phagosome, cell adhesion molecules, and C-type lectin receptor signaling. DEGs annotated to these pathways included *H2-Aa*, *NOS2*, *MR1*, *HLA-DPB1*, and *CLEC10A*. At 14 dpi, tuberculosis, C-type lectin receptor signaling, African trypanosomiasis, hematopoietic cell lineage, and amoebiasis were among the main enriched pathways, involving *NOS2*, *CLEC10A*, *HBB*, *HLA-DPB1*, and *COL3A1*. The comparison between 14 and 7 dpi showed enrichment in malaria, African trypanosomiasis, relaxin signaling, porphyrin metabolism, and toxoplasmosis, with *HBB*, *HBAA1*, *COL3A1*, *FTH1*, and *H2-Aa* among the corresponding DEGs. The enrichment of these KEGG disease pathways likely reflects the presence of shared conserved immune genes and should not be interpreted as evidence that the corresponding diseases occurred in hybrid sturgeon.

**Table 4:** Top KEGG pathways enriched among DEGs.

Tissue	Comparison	KEGG pathway	Pathway *P* value	Pathway FDR	Representative pathway-associated DEG	Gene log₂FC	Gene FDR
Spiral valve intestine	14 dpi vs. 7 dpi	Malaria	8.93E-25	2.83E-22	*HBB*	−4.906	1.38E-17
Spiral valve intestine	14 dpi vs. 7 dpi	African trypanosomiasis	1.46E-13	2.32E-11	*HBAA1*	−4.789	6.83E-13
Spiral valve intestine	14 dpi vs. 7 dpi	Relaxin signaling pathway	4.06E-07	4.29E-05	*COL3A1*	−4.732	3.12E-08
Spiral valve intestine	14 dpi vs. 7 dpi	Porphyrin metabolism	5.93E-07	4.70E-05	*FTH1*	2.819	2.43E-05
Spiral valve intestine	14 dpi vs. 7 dpi	Toxoplasmosis	1.49E-06	9.46E-05	*H2-Aa*	−6.475	5.72E-05
Spiral valve intestine	14 dpi vs. Control	Tuberculosis	1.21E-55	3.75E-53	*NOS2*	4.846	9.76E-11
Spiral valve intestine	14 dpi vs. Control	C-type lectin receptor signaling pathway	9.76E-45	1.51E-42	*CLEC10a*	7.492	7.79E-08
Spiral valve intestine	14 dpi vs. Control	African trypanosomiasis	1.56E-40	1.61E-38	*HBB*	−7.020	6.76E-26
Spiral valve intestine	14 dpi vs. Control	Hematopoietic cell lineage	5.44E-24	4.21E-22	*HLA-DPB1*	−3.198	4.86E-07
Spiral valve intestine	14 dpi vs. Control	Amoebiasis	1.55E-23	9.61E-22	*COL3A1*	−5.060	3.90E-10
Spiral valve intestine	7 dpi vs. Control	Hematopoietic cell lineage	8.35E-55	2.70E-52	*H2-Aa*	−3.944	9.39E-11
Spiral valve intestine	7 dpi vs. Control	Tuberculosis	3.78E-49	6.10E-47	*NOS2*	7.626	1.64E-30
Spiral valve intestine	7 dpi vs. Control	Phagosome	1.26E-17	1.36E-15	*MR1*	−7.262	4.26E-04
Spiral valve intestine	7 dpi vs. Control	Cell adhesion molecules	1.96E-17	1.59E-15	*HLA-DPB1*	−3.749	8.42E-08
Spiral valve intestine	7 dpi vs. Control	C-type lectin receptor signaling pathway	1.32E-16	8.50E-15	*CLEC10a*	5.668	2.99E-06
Liver	14 dpi vs. 7 dpi	Cysteine and methionine metabolism	2.78E-32	9.25E-30	*BHMT*	2.832	1.61E-04
Liver	14 dpi vs. 7 dpi	*Staphylococcus aureus* infection	1.76E-28	2.93E-26	*H2-Aa*	−7.902	1.20E-05
Liver	14 dpi vs. 7 dpi	Porphyrin metabolism	1.45E-27	1.61E-25	*FTH*	4.123	8.80E-08
Liver	14 dpi vs. 7 dpi	Coronavirus disease - COVID-19	2.84E-26	2.37E-24	*CFB*	−7.029	9.13E-09
Liver	14 dpi vs. 7 dpi	Ferroptosis	3.60E-23	2.40E-21	*TF2*	−5.215	5.93E-13
Liver	14 dpi vs. Control	Cysteine and methionine metabolism	7.92E-39	2.80E-36	*TAT*	3.241	2.12E-07
Liver	14 dpi vs. Control	Mineral absorption	5.86E-34	1.03E-31	*HMOX1*	4.357	9.12E-11
Liver	14 dpi vs. Control	Ferroptosis	1.38E-33	1.62E-31	*TF1*	−4.228	4.24E-10
Liver	14 dpi vs. Control	Antigen processing and presentation	5.87E-26	5.18E-24	*HSP90A.1*	5.951	7.02E-07
Liver	14 dpi vs. Control	Protein digestion and absorption	8.20E-23	5.79E-21	*COL3A1*	−3.261	4.14E-08
Liver	7 dpi vs. Control	Tuberculosis	1.12E-42	3.94E-40	*NOS2*	6.851	2.51E-15
Liver	7 dpi vs. Control	Ferroptosis	2.30E-25	4.05E-23	*FTH1*	4.032	1.90E-08
Liver	7 dpi vs. Control	Complement and coagulation cascades	1.52E-23	1.78E-21	*F13B*	13.497	1.01E-10
Liver	7 dpi vs. Control	C-type lectin receptor signaling pathway	7.01E-22	6.17E-20	*CD209*	2.680	9.35E-09
Liver	7 dpi vs. Control	Neutrophil extracellular trap formation	1.21E-21	8.53E-20	*NCF4*	3.458	5.20E-11

Only the top five pathways ranked by *p*-value are shown for each tissue-comparison pair. KEGG disease-pathway names reflect shared conserved genes and do not indicate the occurrence of the corresponding diseases in hybrid sturgeon.

To validate the DEGs identified by RNA-seq, 10 genes from the liver and spiral valve intestine were selected for qRT-PCR confirmation, including *CFB*, *MIF*, *HLA-DPB1*, *CTSL*, *LPL*, *CLEC17A*, *H2-Aa*, *MYD88*, *NOS2* and *FTH1* (Supplementary Figure S2). The candidate reference genes *β-actin*, *GAPDH* and *EF1α* were evaluated for expression stability. The NormFinder stability values for *β-actin*, *EF1α*, and *GAPDH* were 0.224, 0.313, and 1.208, respectively. *β-actin* had the lowest NormFinder stability value and was therefore selected as the reference gene for expression normalization. The qRT-PCR results were then compared with the RNA-seq data using log_2_FC values at 7 and 14 dpi relative to the control group. Across the 20 paired log₂FC values, the RNA-seq and RT-qPCR results were strongly correlated (Pearson r = 0.963, *p* = 1.06 × 10^−11^; Supplementary Figure S2), supporting the consistency between the two methods.

### 3.4 Metabolomic profiles and KEGG enrichment in liver and spiral valve intestine

Untargeted LC–MS metabolomics was used to characterize metabolic profiles in the liver and spiral valve intestine. PCA of metabolomics data showed the distribution of biological samples and pooled QC samples, while Pearson correlation analysis showed overall sample-level consistency within the metabolomics dataset (Supplementary Figure S3). Differential metabolite analysis identified a large number of metabolites that differed among the baseline control, 7-dpi, and 14-dpi groups in both tissues (Figure 5A; Table 5). In the liver, 673 differential metabolites were identified in the 7 dpi vs. Control comparison, including 303 upregulated and 370 downregulated metabolites. At 14 dpi, 798 differential metabolites were detected, with 460 upregulated and 338 downregulated metabolites. The 14 dpi vs. 7 dpi comparison identified 783 differential metabolites, including 519 upregulated and 264 downregulated metabolites. In the spiral valve intestine, 729 differential metabolites were identified in the 7 dpi vs. control comparison, including 329 upregulated and 400 downregulated metabolites. The 14 dpi vs. control comparison detected 776 differential metabolites, with 270 upregulated and 506 downregulated metabolites. Between 14 dpi and 7 dpi, 788 differential metabolites were identified, including 257 upregulated and 531 downregulated metabolites.

**Figure 5:**
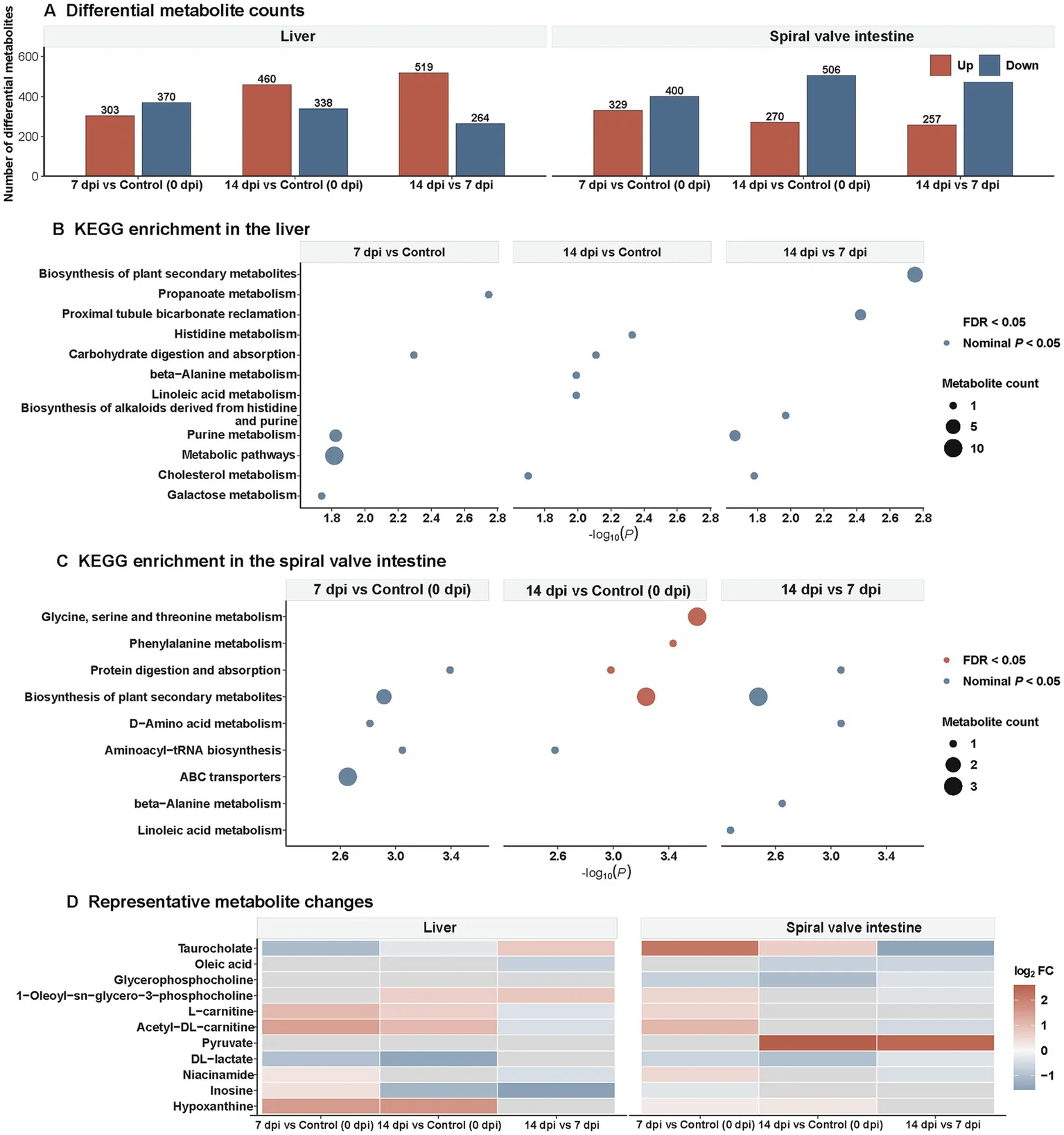
Metabolomic profiles and KEGG enrichment in liver and spiral valve intestine. **(A)** Numbers of upregulated and downregulated differential metabolites in each comparison. Up and down indicate metabolites increased or decreased in the first group relative to the second group. **(B)** KEGG enrichment of differential metabolites in the liver. **(C)** KEGG enrichment of differential metabolites in the spiral valve intestine. Red bubbles indicate pathways with FDR < 0.05, whereas blue bubbles indicate pathways with *p* < 0.05 but FDR ≥ 0.05. **(D)** Fold-change patterns of representative metabolites across tissues and infection stages.

**Table 5:** Differential metabolites in liver and spiral valve intestine.

Tissue	Comparison	Upregulated	Downregulated	Total differential metabolites
Liver	7 dpi vs. Control	303	370	673
Liver	14 dpi vs. Control	460	338	798
Liver	14 dpi vs. 7 dpi	519	264	783
Spiral valve intestine	7 dpi vs. Control	329	400	729
Spiral valve intestine	14 dpi vs. Control	270	506	776
Spiral valve intestine	14 dpi vs. 7 dpi	257	531	788

KEGG enrichment analysis further showed tissue-related differences in metabolomic pathway profiles (Figures 5B,C; Supplementary Table S4). In the liver, enriched pathways were mainly detected at the nominal significance level, including propanoate metabolism, carbohydrate digestion and absorption, purine metabolism, galactose metabolism, histidine metabolism, beta-alanine metabolism, linoleic acid metabolism, and cholesterol metabolism. In the spiral valve intestine, several pathways reached FDR-adjusted significance in the 14 dpi vs. control comparison, including glycine, serine and threonine metabolism, phenylalanine metabolism, biosynthesis of plant secondary metabolites, and protein digestion and absorption. Other pathways, such as D-amino acid metabolism, aminoacyl-tRNA biosynthesis, ABC transporters, beta-alanine metabolism, and linoleic acid metabolism, were also detected among the enriched pathway list across comparisons. Representative metabolites showed tissue- and stage-related changes (Figure 5D; Supplementary Table S5). These metabolites included taurocholate, oleic acid, glycerophosphocholine, 1-oleoyl-sn-glycero-3-phosphocholine, L-carnitine, acetyl-DL-carnitine, pyruvate, DL-lactate, niacinamide, inosine, and hypoxanthine. Their fold-change patterns differed between liver and spiral valve intestine, as well as between 7 dpi and 14 dpi. The tank-level sensitivity analysis showed strong agreement with the individual-fish analysis, with Spearman correlations of log₂ fold changes ranging from 0.967 to 0.981 and directional concordance ranging from 99.86 to 100% (Supplementary Table S6).

### 3.5 Cross-omics comparison and exploratory associations

The transcriptomic and metabolomic results were first compared at the pathway level (Figure 6A). Across the three comparisons, 138–183 KEGG pathways in the spiral valve intestine and 157–164 pathways in the liver contained both DEGs and differential metabolites. However, protein digestion and absorption pathway was the only pathway that reached FDR < 0.05 in both datasets, and this was observed in the spiral valve intestine at 14 dpi compared with the control. No pathway reached FDR < 0.05 in both datasets in the liver. The directional patterns of candidate gene–metabolite pairs were further summarized using nine-quadrant plots (Supplementary Figure S4). Exploratory analysis of the 12 matched intestinal samples identified candidate associations meeting BH-adjusted *p* < 0.10 between bacterial genera, including *Candidatus Arthromitus*, *Clostridioides*, and *Afipia*, and metabolites, including inosine, acetyl-DL-carnitine, L-carnitine, niacinamide, and taurocholate (Figure 6B). Group-adjusted partial Spearman analysis further identified eight gene–metabolite associations in the spiral valve intestine and 16 in the liver (Figures 6C,D). The intestinal network included ALDH6A1, COL3A1, FAH, GNMT, NOS2, PNPLA2, CAHZ, and GALC, together with seven metabolites. The liver network included FPGS, PNPLA2, RBP7, AHCY, CHKA, and CNDP2, together with four metabolites. These associations describe statistical covariation among matched samples and do not demonstrate direct regulation between genes and metabolites. The ITS PERMANOVA result was considered complementary community-level microbiome evidence in the multi-omics interpretation. Because amplicon sequencing does not directly establish fungal metabolic functions, fungal taxa were not assigned to KEGG pathways. Moreover, given the absence of FDR-supported fungal genera and the limited number of matched samples, we did not extend the exploratory genus–metabolite association analysis to fungal taxa.

**Figure 6:**
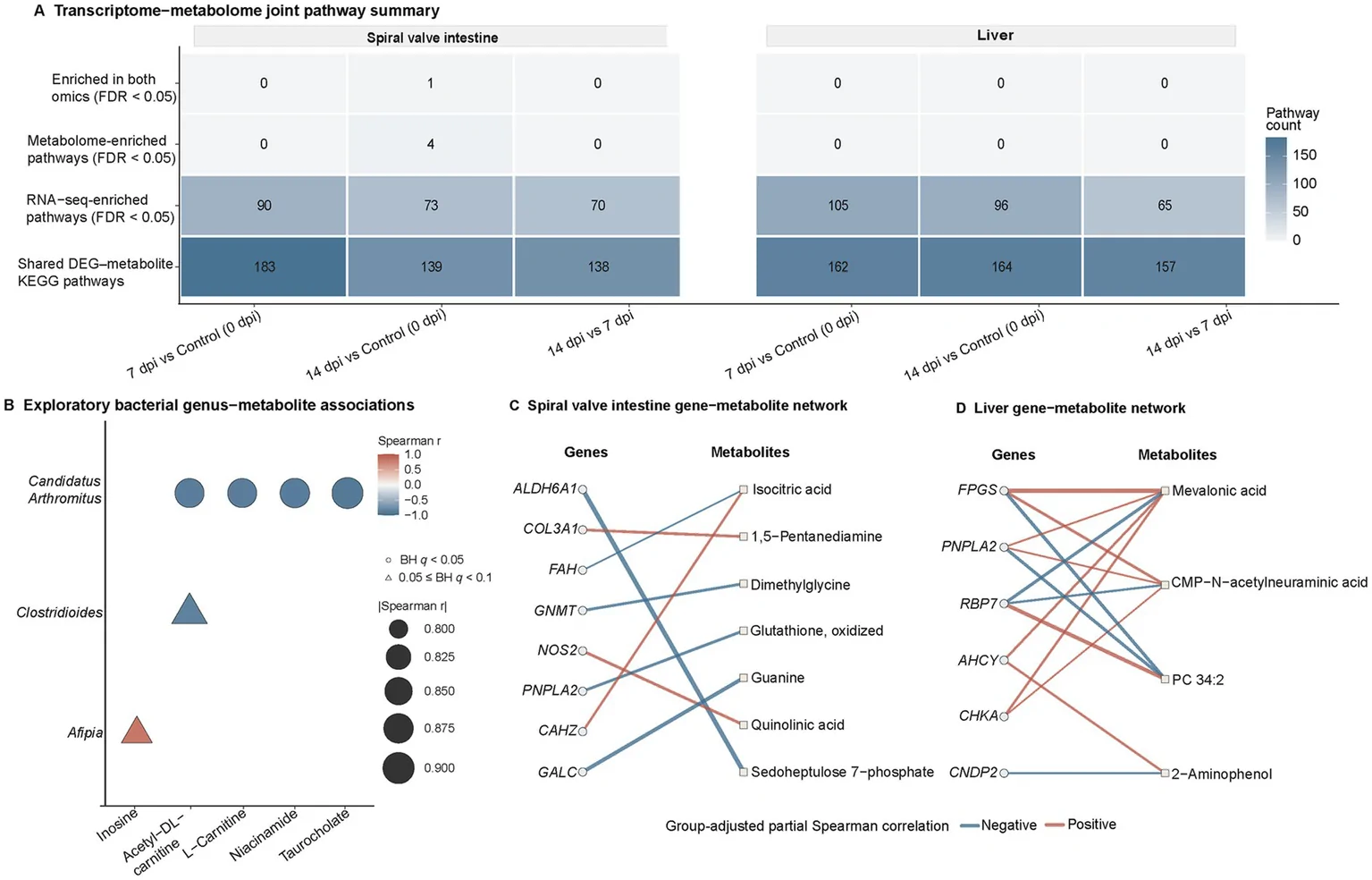
Cross-omics comparison and exploratory associations. **(A)** Pathway-level comparison of the transcriptomic and metabolomic results in the spiral valve intestine and liver. Cell values indicate the numbers of shared DEG–metabolite KEGG pathways, RNA-seq pathways with FDR < 0.05, metabolomic pathways with FDR < 0.05, and pathways reaching FDR < 0.05 in both datasets. **(B)** Exploratory Spearman associations between bacterial genera and representative intestinal metabolites based on 12 matched samples. Point color indicates the correlation coefficient, point size indicates |Spearman ρ|, and point shape indicates the BH-adjusted *p*-value category. Only associations with BH-adjusted *p* < 0.10 are shown. **(C,D)** Gene–metabolite networks in the spiral valve intestine and liver based on group-adjusted partial Spearman correlations. Red and blue lines indicate positive and negative associations, respectively, and line width represents the absolute partial correlation coefficient. Only pairs with shared KEGG annotations, partial *p* < 0.05, and BH-adjusted partial *p* < 0.10 are shown.

## 4 Discussion

### 4.1 Changes in intestinal bacterial and fungal community profiles across sampled groups

The intestine is one of the main sites where microbial communities, dietary components, and mucosal immune responses interact. In fish, intestinal microbiota are closely associated with nutrient utilization, epithelial barrier function, immune homeostasis, and resistance to pathogen invasion (Yukgehnaish et al., 2020; Zhang et al., 2024). Changes in gut microbial profiles have also been reported in several fish species after bacterial infection, including *N. seriolae*-infected hybrid snakehead, *A. hydrophila*-infected common carp, and *S. sciuri*-infected hybrid sturgeon (Zhang et al., 2022; Zhang et al., 2023; Zhou et al., 2024). In the present study, no significant differences were detected in bacterial alpha-diversity indices among the control, 7-dpi, and 14-dpi groups. This result indicates that no marked difference in overall bacterial richness or evenness was detected between the infected groups and the baseline control under the present experimental conditions. Although alpha diversity remained statistically comparable among groups, beta-diversity analysis showed a different pattern. Bray–Curtis and unweighted UniFrac PERMANOVA detected significant differences among groups, whereas PERMDISP tests were not significant. This indicates that the observed group separation was less likely to be driven only by unequal within-group dispersion. Interestingly, weighted UniFrac did not reach significance, while unweighted UniFrac was significant. Since unweighted UniFrac is more sensitive to the presence or absence of taxa, this pattern may reflect differences in low-abundance or phylogenetically distinct bacterial members among the sampled groups rather than large changes in dominant taxa alone.

At the taxonomic level, bacterial relative-abundance profiles varied across the sampled groups. Firmicutes accounted for a larger proportion at 7 dpi, while Proteobacteria showed a lower proportion in the infected groups than in the control group. A similar increase in Firmicutes and decrease in Proteobacteria was reported in hybrid sturgeon infected with *S. sciuri* (Zhang et al., 2022), and *A. hydrophila* infection in common carp also changed the relative abundance of Firmicutes, Bacteroidetes, and Proteobacteria (Zhang et al., 2023). In hybrid snakehead challenged with *N. seriolae*, intestinal microbiota composition differed across the sampled groups and was accompanied by intestinal tissue damage and altered expression of immune-related genes (Zhou et al., 2024). Together with these studies, our data suggest that *N. seriolae* infection was associated with altered intestinal bacterial community profiles in hybrid sturgeon, and that the direction and magnitude of these changes may vary with host species, pathogen type, and infection stage.

Several dominant genera also showed different abundance patterns across infection stages, including *Sarcina*, *Clostridium*, *Acinetobacter*, *Gloeocapsa*, *Variovorax*, *Bradyrhizobium*, and *Candidatus Arthromitus*. Some of these taxa have been reported in diseased or stressed sturgeons. For example, *Candidatus Arthromitus* was markedly enriched in *S. sciuri*-infected hybrid sturgeon, while changes in *Clostridium*, *Cetobacterium*, *Lactococcus*, and *Mycoplasma* were observed in hybrid sturgeon with enteritis associated with intestinal microbiota disorder (Huang et al., 2020; Zhang et al., 2022). However, genus-level changes based on 16S data should be interpreted carefully, because relative abundance patterns alone do not indicate whether a taxon is beneficial, opportunistic, or directly involved in disease progression. Overall, our results indicate that *N. seriolae* infection was associated with intestinal bacterial community restructuring, even though a generalized decrease in bacterial alpha diversity was not observed.

The ITS data provide a complementary view of intestinal microbial variation across the sampled stages. Significant overall differences in fungal richness and diversity, together with the PERMANOVA result, indicate that the fungal component of the intestinal microbiota also varied among groups. However, these community-level patterns were not attributable to individual fungal genera after FDR correction. Although Fusarium, Kazachstania, Aspergillus, Pichia, and several other genera showed different relative-abundance patterns among samples, these descriptive changes do not establish taxon-specific responses or functional roles.

### 4.2 Transcriptional responses in the liver and spiral valve intestine

The liver showed a larger number of DEGs than the spiral valve intestine at both infection stages, suggesting that hepatic transcriptional responses were more extensive under the present challenge conditions. As a central organ for metabolic and immune regulation, the liver contributes to acute-phase responses, complement activity, detoxification, lipid metabolism, and redox homeostasis (Bayne and Gerwick, 2001). Similar hepatic or systemic immune responses have been reported in other fish after bacterial infection. In Russian sturgeon, *A. hydrophila* challenge altered hepatic transcriptional profiles related to inflammatory responses and metabolic regulation (Costábile et al., 2022). In largemouth bass infected with *N. seriolae*, integrated transcriptomic and metabolomic analyses also suggested that immune responses were accompanied by changes in carbohydrate, amino acid, and energy-related pathways (Dong et al., 2024). Together with these studies, our results suggest that the liver may be an important site for systemic immune and immunometabolic changes following experimental *N. seriolae* challenge.

In the liver, DEGs were enriched in pathways associated with complement and coagulation cascades, C-type lectin receptor signaling, neutrophil extracellular trap formation, antigen processing and presentation, cysteine and methionine metabolism, porphyrin metabolism, and ferroptosis-related processes. Complement and coagulation cascades have frequently been reported in sturgeon bacterial infection studies. For example, hybrid sturgeon infected with *A. hydrophila* showed upregulation of genes associated with Toll-like receptor signaling, NF-κB signaling, phagocytosis, mannose receptor-mediated recognition, and complement pathways (Jiang et al., 2018). In Dabry’s sturgeon, *A. hydrophila* infection induced head-kidney transcriptional changes involving Toll-like receptor signaling, NOD-like receptor signaling, RIG-I-like receptor signaling, chemokine signaling, and complement and coagulation cascades (Luo et al., 2018). Similarly, *Y. ruckeri* infection in Amur sturgeon affected pathways such as antigen processing and presentation, complement and coagulation cascades, T-cell receptor signaling, B-cell receptor signaling, NOD-like receptor signaling, and chemokine signaling (Li et al., 2017). These reports support the view that complement activity, pathogen recognition, and antigen-related processes are common components of bacterial infection responses in sturgeons.

The enrichment of C-type lectin receptor signaling and phagocytosis-related pathways may reflect changes in pathogen recognition and cellular immune processes. *N. seriolae* is an intracellularly persistent bacterial pathogen in fish, and studies in snakehead and largemouth bass have suggested that infection can induce immune pathways related to pattern recognition, phagocytosis, cytokine signaling, cell death, and granulomatous inflammation (Chen J. et al., 2018; Dong et al., 2024; Liu et al., 2022). Our previous study on naturally infected hybrid sturgeon also showed marked pathological lesions and spleen transcriptomic changes involving immune-related pathways, although that case involved co-detection of white sturgeon iridovirus (Ni et al., 2024). In the present experimental model, the enrichment of complement, lectin receptor, neutrophil-related, and antigen-presentation pathways was consistent with a broad hepatic immune transcriptional response following *N. seriolae* challenge.

The spiral valve intestine showed fewer DEGs than the liver, but its enriched pathways were still closely related to local immune processes. DEGs in this tissue were enriched in phagosome, cell adhesion molecules, hematopoietic cell lineage, C-type lectin receptor signaling pathway, and several infection-related KEGG pathways. These results may indicate transcriptional adjustment of mucosal immune recognition, immune-cell-related processes, phagocytosis, and epithelial or cell-interaction pathways in the spiral valve intestine. Similar intestinal immune responses have been reported in other fish bacterial infection models. In Chinese sea bass infected with *A. hydrophila*, intestinal transcriptomic analysis showed enrichment of NOD-like receptor, Toll-like receptor, and C-type lectin receptor signaling pathways, together with clear changes in intestinal microbial composition and tissue morphology (Pan et al., 2023). In grass carp, *A. hydrophila* infection also induced marked changes in intestine inflammation-related genes (Song et al., 2017). These findings are broadly consistent with our observation that the spiral valve intestine participated in the host response, although the overall DEG scale was smaller than that in the liver.

### 4.3 Metabolic changes across sampled groups and tissues

Metabolic changes were detected among the sampled groups in both the liver and spiral valve intestine, indicating tissue- and stage-related metabolic variation following experimental challenge. However, the pathway-level evidence was not evenly distributed between tissues. In the spiral valve intestine, glycine, serine and threonine metabolism, phenylalanine metabolism, biosynthesis of plant secondary metabolites, and protein digestion and absorption reached FDR-adjusted significance when the 14-dpi group was compared with the control group. By contrast, most liver metabolic pathways showed *p* < 0.05 but did not remain significant after FDR correction. Thus, the metabolomics data showed clearer intestinal metabolic differences at 14 dpi relative to the baseline control, whereas the liver pathway results should be interpreted as suggestive signals.

Amino acid metabolism was one of the main metabolic themes in the spiral valve intestine. Similar amino-acid-related changes have been reported in fish bacterial infection models. In *N. seriolae*-infected largemouth bass, transcriptome–metabolome integration identified changes in glycolysis, the TCA cycle, amino acid metabolism, and arginine-related pathways (Dong et al., 2024). A liver-focused study in largemouth bass also suggested that *N. seriolae* infection affected arginine metabolism, glutathione metabolism, and antioxidant defense (Sun et al., 2025). In Nile tilapia, serine metabolism was linked to host survival after *Edwardsiella tarda* infection, partly through ROS and glutathione-related metabolism (Yang D. et al., 2021). In common carp infected with *A. hydrophila*, intestinal multi-omics analysis also showed amino acid metabolism changes together with immune-related pathways (Zhang et al., 2023). These studies provide a useful context for our data and suggest that the amino-acid-related changes observed in the spiral valve intestine may be associated with the sampled stages following *N. seriolae* challenge.

Representative metabolites, including taurocholate, oleic acid, glycerophosphocholine, L-carnitine, acetyl-DL-carnitine, pyruvate, DL-lactate, inosine, hypoxanthine, and niacinamide, further showed tissue- and stage-related variation. These metabolites are broadly related to lipid and bile acid metabolism, carnitine-associated lipid utilization, glycolytic end products, and purine/nicotinamide metabolism. Bile acids are involved in lipid digestion and also interact with intestinal microbiota and host metabolic regulation (Wahlström et al., 2016). In fish, intestinal microbiota has been reported to participate in bile acid metabolism, and bile acid-related regulation may influence hepatic lipid metabolism and metabolic health (Luo et al., 2023; Wang et al., 2023; Xiong et al., 2022). In the present study, these metabolite patterns do not define a single metabolic mechanism, but they indicate concurrent intestinal and hepatic metabolic variation associated with experimental *N. seriolae* challenge.

### 4.4 Genus–metabolite associations between intestinal bacteria and metabolic features

The genus–metabolite association analysis was used to identify candidate links between intestinal bacterial profiles and metabolite variation across the sampled groups. Similar integrated analyses have been used in fish bacterial infection studies to connect altered gut microbial communities with host metabolic responses. In common carp, *A. hydrophila* infection changed intestinal microbiota, immune-related transcriptomic pathways, and metabolomic profiles, with amino acid metabolism emerging as one of the main affected metabolic processes (Zhang et al., 2023). In largemouth bass challenged with *A. hydrophila*, intestinal microbiota variation was analyzed together with serum metabolomics, and several infection-related microbiota-associated metabolite markers were identified (Jin et al., 2023). These studies suggest that microbe–metabolite association analysis can help narrow down potential microbial and metabolic features involved in host responses to bacterial infection.

In the present study, several bacterial genera, including *Sarcina*, *Clostridium*, *Acinetobacter*, *Staphylococcus*, *Novosphingobium*, and *Candidatus Arthromitus*, were associated with representative intestinal metabolites such as taurocholate, glycerophosphocholine, L-carnitine, acetyl-DL-carnitine, pyruvate, inosine, hypoxanthine, and niacinamide. These metabolites were mainly related to lipid and bile acid metabolism, carnitine-associated lipid utilization, energy metabolism, and purine/nicotinamide metabolism. Because no bacterial genus showed FDR-supported differential abundance, these genus–metabolite associations were treated as exploratory, and the analysis was not extended to a gene–bacterial genus network. Together with the bacterial beta-diversity results and metabolomic pathway enrichment, these associations suggest that differences in intestinal bacterial community profiles across the sampled groups occurred alongside variation in several metabolic features. These results are better suited for prioritizing candidate genus–metabolite pairs for future validation and provide clues for further examining the relationship between intestinal microbial changes and metabolic changes following experimental challenge. Overall, the multi-omics results suggest that *N. seriolae* infection in hybrid sturgeon was associated with intestinal bacterial community restructuring, broader hepatic transcriptional responses than those observed in the spiral valve intestine, and metabolic changes related to tissue type and sampling stage. The genus–metabolite associations further linked part of the microbial variation with metabolites involved in lipid, bile acid, energy, and purine/nicotinamide-related metabolism. These findings provide a basis for future targeted studies on microbial and metabolic features associated with nocardial infection in hybrid sturgeons.

The clinical signs, gross lesions, and successful re-isolation of *N. seriolae* confirmed that the experimental infection was established. However, the PBS control fish were sampled only at 0 dpi, without time-matched controls at 7 or 14 dpi. Thus, although the comparisons identified multi-omics changes occurring after challenge, they could not fully separate infection-associated changes from other variation over time, particularly in the microbiome and metabolome. Potential within-tank dependence was also considered in the metabolomic analysis. The tank-level sensitivity analysis supported the overall directions of the metabolite changes, although such dependence cannot be completely excluded. Accordingly, the multi-omics findings were interpreted as changes associated with experimental challenge rather than effects caused solely by *N. seriolae* infection.

## 5 Conclusion

In this study, microbiome, transcriptome, and metabolome profiling was used to characterize changes in hybrid sturgeon following experimental *N. seriolae* challenge. Compared with the baseline control group, fish sampled at 7 and 14 dpi showed differences in intestinal bacterial community composition, whereas bacterial alpha diversity did not differ significantly among the three sampled groups. Fungal diversity and community composition also differed among the sampled groups, although no fungal genus remained significant after FDR correction. RNA-seq analysis revealed tissue-specific transcriptional responses, with the liver showing a larger number of DEGs and broader enrichment of immune- and stress-related pathways than the spiral valve intestine. Metabolomic analysis indicated metabolic changes related to tissue type and sampling stage, mainly involving amino acid metabolism, lipid and bile acid metabolism, energy metabolism, and purine/nicotinamide-related processes. Genus–metabolite association analysis further identified candidate relationships between intestinal bacterial genera and representative metabolites. These findings provide a multi-omics basis for understanding intestinal microbial and tissue-specific immunometabolic changes associated with experimental *N. seriolae* challenge in hybrid sturgeon.

## Data Availability

The metabolomics data reported in this study have been deposited in OMIX, China National Center for Bioinformation/Beijing Institute of Genomics, Chinese Academy of Sciences (https://ngdc.cncb.ac.cn/omix; accession number OMIX020370). The bacterial full-length 16S rRNA gene and fungal ITS amplicon sequencing data from intestinal samples, the Illumina RNA-seq data from liver and spiral valve intestine tissues, and the PacBio full-length transcriptome data have been deposited in the Genome Sequence Archive (GSA), National Genomics Data Center, China National Center for Bioinformation, under BioProject accession number PRJCA070388 (https://ngdc.cncb.ac.cn/gsa/).
