# Supplementary material for: Multi-omics profiling identifies intestinal microbial shifts and tissue-specific immunometabolic changes associated with experimental Nocardia seriolae challenge in hybrid sturgeon (Acipenser baerii♀ × Acipenser schrenckii♂)
Source: Front Microbiol. 2026 Sep 18;17:1956937. doi: 10.3389/fmicb.2026.1956937 (PMC13631057; doi:10.3389/fmicb.2026.1956937)
Supplement: Supplementary file 1 [file Data_Sheet_1.docx]

**Figure S1 Quality assessment of RNA-seq samples.** (A) Principal component analysis (PCA) of RNA-seq samples from liver and spiral valve intestine. (B) Pearson correlation heatmap based on gene expression profiles. Li, liver; Int, spiral valve intestine.

**Figure S2. qRT-PCR validation of selected genes from the RNA-seq dataset.** (A) Comparison of RNA-seq and qRT-PCR results for ten genes at 7 and 14 dpi relative to the control group. The heatmap shows log_2_FC values; red indicates up-regulation and blue indicates down-regulation. (B) Scatterplot showing the correlation between RNA-seq and qRT-PCR data.

**Figure S3. Quality assessment of metabolomics samples.** (A) PCA of liver and spiral valve intestine metabolomics samples, including pooled QC samples. (B) Pearson correlation heatmap based on metabolite abundance profiles. Li, liver; Int, spiral valve intestine; QC, quality control sample.

**Figure S4. Nine-quadrant analysis of transcriptome–metabolome fold-change patterns. (**A–C) Spiral valve intestine. (D–F) Liver. Each point represents a candidate gene–metabolite pair. The x- and y-axes show metabolite and gene log₂FC values, respectively. Dashed lines indicate |log₂FC| = 1. Red and blue points indicate concordant increases and decreases, respectively; yellow points indicate opposite directions, and gray points indicate that one or both features had |log₂FC| < 1.

| **Supplementary Table S1.** **Sample allocation.** | | | | | | | | |
| --- | --- | --- | --- | --- | --- | --- | --- | --- |
| **Group** | **Sampling time** | **Tank ID** | **Fish ID** | **Sample material** | **16S sample ID** | **ITS sample ID** | **RNA-seq sample ID** | **Metabolomics sample ID** |
| PBS baseline control | 0 dpi | Tank 1 | C-1 | Intestinal contents | XZ-C-1 | XZ-C-1 | — | — |
| PBS baseline control | 0 dpi | Tank 2 | C-2 | Intestinal contents | XZ-C-2 | XZ-C-2 | — | — |
| PBS baseline control | 0 dpi | Tank 3 | C-3 | Intestinal contents | XZ-C-3 | XZ-C-3 | — | — |
| PBS baseline control | 0 dpi | Tank 4 | C-4 | Intestinal contents | XZ-C-4 | XZ-C-4 | — | — |
| PBS baseline control | 0 dpi | Tank 1 | C-1 | Liver | — | — | XZ-C-Li1 | XZ-C-Li1 |
| PBS baseline control | 0 dpi | Tank 2 | C-2 | Liver | — | — | XZ-C-Li2 | XZ-C-Li2 |
| PBS baseline control | 0 dpi | Tank 3 | C-3 | Liver | — | — | XZ-C-Li3 | XZ-C-Li3 |
| PBS baseline control | 0 dpi | Tank 4 | C-4 | Liver | — | — | XZ-C-Li4 | XZ-C-Li4 |
| PBS baseline control | 0 dpi | Tank 3 | C-5 | Liver | — | — | — | XZ-C-Li5 |
| PBS baseline control | 0 dpi | Tank 4 | C-6 | Liver | — | — | — | XZ-C-Li6 |
| PBS baseline control | 0 dpi | Tank 1 | C-1 | Spiral valve intestine | — | — | XZ-C-Sp1 | XZ-C-Sp1 |
| PBS baseline control | 0 dpi | Tank 2 | C-2 | Spiral valve intestine | — | — | XZ-C-Sp2 | XZ-C-Sp2 |
| PBS baseline control | 0 dpi | Tank 3 | C-3 | Spiral valve intestine | — | — | XZ-C-Sp3 | XZ-C-Sp3 |
| PBS baseline control | 0 dpi | Tank 4 | C-4 | Spiral valve intestine | — | — | XZ-C-Sp4 | XZ-C-Sp4 |
| PBS baseline control | 0 dpi | Tank 3 | C-5 | Spiral valve intestine | — | — | — | XZ-C-Sp5 |
| PBS baseline control | 0 dpi | Tank 4 | C-6 | Spiral valve intestine | — | — | — | XZ-C-Sp6 |
| *N. seriolae*-infected | 7 dpi | Tank 1 | 7D-1 | Intestinal contents | XZ-7D-1 | XZ-7D-1 | — | — |
| *N. seriolae*-infected | 7 dpi | Tank 2 | 7D-2 | Intestinal contents | XZ-7D-2 | XZ-7D-2 | — | — |
| *N. seriolae*-infected | 7 dpi | Tank 3 | 7D-3 | Intestinal contents | XZ-7D-3 | XZ-7D-3 | — | — |
| *N. seriolae*-infected | 7 dpi | Tank 4 | 7D-4 | Intestinal contents | XZ-7D-4 | XZ-7D-4 | — | — |
| *N. seriolae*-infected | 7 dpi | Tank 1 | 7D-1 | Liver | — | — | XZ-7d-Li1 | XZ-7d-Li1 |
| *N. seriolae*-infected | 7 dpi | Tank 2 | 7D-2 | Liver | — | — | XZ-7d-Li2 | XZ-7d-Li2 |
| *N. seriolae*-infected | 7 dpi | Tank 3 | 7D-3 | Liver | — | — | XZ-7d-Li3 | XZ-7d-Li3 |
| *N. seriolae*-infected | 7 dpi | Tank 4 | 7D-4 | Liver | — | — | XZ-7d-Li4 | XZ-7d-Li4 |
| *N. seriolae*-infected | 7 dpi | Tank 3 | 7D-5 | Liver | — | — | — | XZ-7d-Li5 |
| *N. seriolae*-infected | 7 dpi | Tank 4 | 7D-6 | Liver | — | — | — | XZ-7d-Li6 |
| *N. seriolae*-infected | 7 dpi | Tank 1 | 7D-1 | Spiral valve intestine | — | — | XZ-7d-Sp1 | XZ-7d-Sp1 |
| N. seriolae-infected | 7 dpi | Tank 2 | 7D-2 | Spiral valve intestine | — | — | XZ-7d-Sp2 | XZ-7d-Sp2 |
| *N. seriolae*-infected | 7 dpi | Tank 3 | 7D-3 | Spiral valve intestine | — | — | XZ-7d-Sp3 | XZ-7d-Sp3 |
| *N. seriolae*-infected | 7 dpi | Tank 4 | 7D-4 | Spiral valve intestine | — | — | XZ-7d-Sp4 | XZ-7d-Sp4 |
| *N. seriolae*-infected | 7 dpi | Tank 3 | 7D-5 | Spiral valve intestine | — | — | — | XZ-7d-Sp5 |
| *N. seriolae*-infected | 7 dpi | Tank 4 | 7D-6 | Spiral valve intestine | — | — | — | XZ-7d-Sp6 |
| *N. seriolae*-infected | 14 dpi | Tank 1 | 14D-1 | Intestinal contents | XZ-14D-1 | XZ-14D-1 | — | — |
| *N. seriolae*-infected | 14 dpi | Tank 2 | 14D-2 | Intestinal contents | XZ-14D-2 | XZ-14D-2 | — | — |
| *N. seriolae*-infected | 14 dpi | Tank 3 | 14D-3 | Intestinal contents | XZ-14D-3 | XZ-14D-3 | — | — |
| *N. seriolae*-infected | 14 dpi | Tank 4 | 14D-4 | Intestinal contents | XZ-14D-4 | XZ-14D-4 | — | — |
| *N. seriolae*-infected | 14 dpi | Tank 1 | 14D-1 | Liver | — | — | XZ-14d-Li1 | XZ-14d-Li1 |
| *N. seriolae*-infected | 14 dpi | Tank 2 | 14D-2 | Liver | — | — | XZ-14d-Li2 | XZ-14d-Li2 |
| *N. seriolae*-infected | 14 dpi | Tank 3 | 14D-3 | Liver | — | — | XZ-14d-Li3 | XZ-14d-Li3 |
| *N. seriolae*-infected | 14 dpi | Tank 4 | 14D-4 | Liver | — | — | XZ-14d-Li4 | XZ-14d-Li4 |
| *N. seriolae*-infected | 14 dpi | Tank 3 | 14D-5 | Liver | — | — | — | XZ-14d-Li5 |
| *N. seriolae*-infected | 14 dpi | Tank 4 | 14D-6 | Liver | — | — | — | XZ-14d-Li6 |
| *N. seriolae*-infected | 14 dpi | Tank 1 | 14D-1 | Spiral valve intestine | — | — | XZ-14d-Sp1 | XZ-14d-Sp1 |
| *N. seriolae*-infected | 14 dpi | Tank 2 | 14D-2 | Spiral valve intestine | — | — | XZ-14d-Sp2 | XZ-14d-Sp2 |
| *N. seriolae*-infected | 14 dpi | Tank 3 | 14D-3 | Spiral valve intestine | — | — | XZ-14d-Sp3 | XZ-14d-Sp3 |
| *N. seriolae*-infected | 14 dpi | Tank 4 | 14D-4 | Spiral valve intestine | — | — | XZ-14d-Sp4 | XZ-14d-Sp4 |
| *N. seriolae*-infected | 14 dpi | Tank 3 | 14D-5 | Spiral valve intestine | — | — | — | XZ-14d-Sp5 |
| *N. seriolae*-infected | 14 dpi | Tank 4 | 14D-6 | Spiral valve intestine | — | — | — | XZ-14d-Sp6 |

**Supplementary Table S2. Gene-specific primers for qRT-PCR validation.**

| **Gene** | **Primer** | **Sequence (5′ to 3′)** | **Product size (bp)** |
| --- | --- | --- | --- |
| *CFB* | F | GCCATGCCTTCCTACAGCATTGTG | 140 |
|  | R | GCATAGGTACCAACAGGGCAGATG |  |
| *MIF* | F | GTCAGAAGCCACACAGGAACTGTC | 139 |
|  | R | CCCAATGCTGTGCAGAGAGCATAG |  |
| *HLA-DPB1* | F | GAAGTTTGTCGGGAACACAGCCTG | 127 |
|  | R | CGATTGTGCCAGGCTTGTAGAGTC |  |
| *CTSL* | F | TTGAACCCAACTTCCTGGAGGCAC | 141 |
|  | R | TCCTGAAGTGCTGACCCTCCAAAG |  |
| *LPL* | F | TGAAGTCCAGGACATTGCCCTCTC | 139 |
|  | R | CCAGCCGAAGTACGAATCCTTCTC |  |
| *CLEC17A* | F | GAGCCAGTTCTGCCCTATCTGAAG | 140 |
|  | R | CTCTGACCAGCGTTCCTGTACAAG |  |
| *H2-Aa* | F | GGAGTCAGCCTCACCCAGTATTAC | 139 |
|  | R | ACGTGGTGAGTGTTTCAGGCAGAG |  |
| *MYD88* | F | TGCTACTGCCACAGGGATATCGAC | 135 |
|  | R | ATGAGTTCGCTGGTGATGCTCCAC |  |
| *NOS2* | F | TTCTACTCCATCAGCTCCTCCCTG | 140 |
|  | R | TCCTTGATGGTGTTCAGCCAGGTG |  |
| *FTH1* | F | CTCCTACGTGTACCTGTCCATGTC | 141 |
|  | R | CCTCTCTGGTTCTGCTGCTTCATC |  |
| *β-actin* | F | ATCCGCAAAGACCTGTACGCCAAC | 140 |
|  | R | CGCTCAGGAGGAGCAATGATCTTG |  |
| *EF1α* | F | AGGCTGACTGTGCTGTGCTGATTG | 140 |
|  | R | CTTGTTGACGCCGATGATGAGCTG |  |

**Supplementary Table S3. Dominant fungal genera inferred from ITS sequencing.**

| **Genus** | **Control** | **7 dpi** | **14 dpi** |
| --- | --- | --- | --- |
| Alternaria | 0.02 | 1.49 | 4.47 |
| Aspergillus | 3.22 | 11.92 | 27.72 |
| Cladosporium | 0.76 | 1.22 | 0.17 |
| Cylindrium | 0.02 | 8.62 | 9.00 |
| Cyphellophora | 2.43 | 0.00 | 0.07 |
| Exophiala | 9.47 | 2.95 | 3.92 |
| Fusarium | 76.68 | 26.23 | 21.69 |
| Geotrichum | 0.01 | 1.35 | 5.11 |
| Kazachstania | 0.19 | 37.95 | 0.14 |
| Millerozyma | 0.00 | 2.85 | 3.07 |
| Pichia | 0.01 | 0.09 | 10.16 |
| Xeromyces | 0.19 | 0.82 | 4.04 |

Note. Values represent mean relative abundance (%) across four fish per group.

**Supplementary Table S4. Top KEGG pathways from metabolomics analysis.**

| **Tissue** | **Comparison** | **KEGG pathway** | ***P* value** | **FDR** |
| --- | --- | --- | --- | --- |
| Spiral valve intestine | 14 dpi vs 7 dpi | D-Amino acid metabolism | 8.45e-04 | 0.076 |
| Spiral valve intestine | 14 dpi vs 7 dpi | Protein digestion and absorption | 8.46e-04 | 0.076 |
| Spiral valve intestine | 14 dpi vs 7 dpi | beta-Alanine metabolism | 0.002 | 0.135 |
| Spiral valve intestine | 14 dpi vs 7 dpi | Biosynthesis of plant secondary metabolites | 0.003 | 0.136 |
| Spiral valve intestine | 14 dpi vs 7 dpi | Linoleic acid metabolism | 0.005 | 0.136 |
| Spiral valve intestine | 14 dpi vs Control | Glycine, serine and threonine metabolism | 2.48e-04 | 0.035 |
| Spiral valve intestine | 14 dpi vs Control | Phenylalanine metabolism | 3.70e-04 | 0.035 |
| Spiral valve intestine | 14 dpi vs Control | Biosynthesis of plant secondary metabolites | 5.79e-04 | 0.036 |
| Spiral valve intestine | 14 dpi vs Control | Protein digestion and absorption | 0.001 | 0.049 |
| Spiral valve intestine | 14 dpi vs Control | Aminoacyl-tRNA biosynthesis | 0.003 | 0.099 |
| Spiral valve intestine | 7 dpi vs Control | Protein digestion and absorption | 4.05e-04 | 0.085 |
| Spiral valve intestine | 7 dpi vs Control | Aminoacyl-tRNA biosynthesis | 8.93e-04 | 0.085 |
| Spiral valve intestine | 7 dpi vs Control | Biosynthesis of plant secondary metabolites | 0.001 | 0.085 |
| Spiral valve intestine | 7 dpi vs Control | D-Amino acid metabolism | 0.002 | 0.085 |
| Spiral valve intestine | 7 dpi vs Control | ABC transporters | 0.002 | 0.098 |
| Liver | 14 dpi vs 7 dpi | Biosynthesis of plant secondary metabolites | 0.002 | 0.336 |
| Liver | 14 dpi vs 7 dpi | Proximal tubule bicarbonate reclamation | 0.004 | 0.358 |
| Liver | 14 dpi vs 7 dpi | Biosynthesis of alkaloids derived from histidine and purine | 0.011 | 0.571 |
| Liver | 14 dpi vs 7 dpi | Cholesterol metabolism | 0.017 | 0.571 |
| Liver | 14 dpi vs 7 dpi | Purine metabolism | 0.022 | 0.571 |
| Liver | 14 dpi vs Control | Histidine metabolism | 0.005 | 0.482 |
| Liver | 14 dpi vs Control | Carbohydrate digestion and absorption | 0.008 | 0.482 |
| Liver | 14 dpi vs Control | beta-Alanine metabolism | 0.010 | 0.482 |
| Liver | 14 dpi vs Control | Linoleic acid metabolism | 0.010 | 0.482 |
| Liver | 14 dpi vs Control | Cholesterol metabolism | 0.020 | 0.598 |
| Liver | 7 dpi vs Control | Propanoate metabolism | 0.002 | 0.333 |
| Liver | 7 dpi vs Control | Carbohydrate digestion and absorption | 0.005 | 0.471 |
| Liver | 7 dpi vs Control | Purine metabolism | 0.015 | 0.664 |
| Liver | 7 dpi vs Control | Metabolic pathways | 0.015 | 0.664 |
| Liver | 7 dpi vs Control | Galactose metabolism | 0.018 | 0.664 |

Note. Pathways with Q value less than 0.05 are FDR-supported; pathways with P value less than 0.05 but Q value greater than or equal to 0.05 should be treated as nominal exploratory signals.

**Supplementary Table S5. Representative differential metabolites selected for biological interpretation.**

| **Tissue** | **Comparison** | **Metabolite** | **VIP** | **P value** | **FDR** | **log2FC** | **Pathway** |
| --- | --- | --- | --- | --- | --- | --- | --- |
| Spiral valve intestine | 14 dpi vs 7 dpi | Taurocholate | 36.3 | 7.90e-14 | 4.52e-12 | -1.481 | ko01100//Metabolic pathways;ko04976//Bile secreti… |
| Spiral valve intestine | 14 dpi vs 7 dpi | Oleic acid | 12.4 | 1.56e-08 | 1.14e-07 | -0.628 | ko01060//Biosynthesis of plant secondary metaboli… |
| Spiral valve intestine | 14 dpi vs 7 dpi | Pyruvate | 12.3 | 9.29e-06 | 3.29e-05 | 2.441 | ko01100//Metabolic pathways;ko01110//Biosynthesis… |
| Spiral valve intestine | 14 dpi vs 7 dpi | Niacinamide | 9.727 | 7.70e-07 | 3.53e-06 | -0.428 | ko01100//Metabolic pathways;ko01240//Biosynthesis… |
| Spiral valve intestine | 14 dpi vs 7 dpi | Glycerophosphocholine | 9.454 | 1.93e-10 | 2.68e-09 | -0.372 | ko00564//Glycerophospholipid metabolism;ko05231//… |
| Spiral valve intestine | 14 dpi vs 7 dpi | Acetyl-DL-carnitine | 9.084 | 9.12e-15 | 9.23e-13 | -0.524 | ko04931//Insulin resistance |
| Spiral valve intestine | 14 dpi vs 7 dpi | Dl-lactate | 7.178 | 2.18e-12 | 6.62e-11 | -0.346 | - |
| Spiral valve intestine | 14 dpi vs Control | Glycerophosphocholine | 17.3 | 4.53e-17 | 1.77e-14 | -1.064 | ko00564//Glycerophospholipid metabolism;ko05231//… |
| Spiral valve intestine | 14 dpi vs Control | Taurocholate | 15.7 | 8.72e-12 | 1.66e-10 | 0.674 | ko01100//Metabolic pathways;ko04976//Bile secreti… |
| Spiral valve intestine | 14 dpi vs Control | Dl-lactate | 12.9 | 5.46e-17 | 2.03e-14 | -0.990 | - |
| Spiral valve intestine | 14 dpi vs Control | Oleic acid | 12.3 | 1.02e-11 | 1.90e-10 | -0.679 | ko01060//Biosynthesis of plant secondary metaboli… |
| Spiral valve intestine | 14 dpi vs Control | Pyruvate | 11.8 | 6.64e-06 | 2.26e-05 | 2.598 | ko01100//Metabolic pathways;ko01110//Biosynthesis… |
| Spiral valve intestine | 14 dpi vs Control | Hypoxanthine | 9.930 | 2.03e-09 | 1.73e-08 | 0.286 | ko01100//Metabolic pathways;ko00230//Purine metab… |
| Spiral valve intestine | 7 dpi vs Control | Taurocholate | 45.4 | 9.10e-15 | 1.20e-12 | 2.155 | ko01100//Metabolic pathways;ko04976//Bile secreti… |
| Spiral valve intestine | 7 dpi vs Control | Glycerophosphocholine | 17.8 | 3.91e-14 | 3.54e-12 | -0.692 | ko00564//Glycerophospholipid metabolism;ko05231//… |
| Spiral valve intestine | 7 dpi vs Control | Acetyl-DL-carnitine | 13.5 | 5.86e-18 | 7.09e-15 | 1.055 | ko04931//Insulin resistance |
| Spiral valve intestine | 7 dpi vs Control | Dl-lactate | 13.3 | 6.04e-16 | 1.58e-13 | -0.643 | - |
| Spiral valve intestine | 7 dpi vs Control | Niacinamide | 12.1 | 1.49e-07 | 1.10e-06 | 0.517 | ko01100//Metabolic pathways;ko01240//Biosynthesis… |
| Spiral valve intestine | 7 dpi vs Control | Hypoxanthine | 10.5 | 4.89e-08 | 4.15e-07 | 0.230 | ko01100//Metabolic pathways;ko00230//Purine metab… |
| Spiral valve intestine | 7 dpi vs Control | L-Carnitine | 9.075 | 3.68e-14 | 3.39e-12 | 0.552 | ko04976//Bile secretion;ko04714//Thermogenesis;ko… |
| Spiral valve intestine | 7 dpi vs Control | Inosine | 7.954 | 2.19e-10 | 4.10e-09 | -0.320 | ko01100//Metabolic pathways;ko02010//ABC transpor… |
| Liver | 14 dpi vs 7 dpi | Taurocholate | 26.6 | 1.04e-13 | 4.03e-12 | 0.776 | ko01100//Metabolic pathways;ko04976//Bile secreti… |
| Liver | 14 dpi vs 7 dpi | Inosine | 16.5 | 2.41e-09 | 1.90e-08 | -1.560 | ko01100//Metabolic pathways;ko02010//ABC transpor… |
| Liver | 14 dpi vs 7 dpi | Niacinamide | 12.2 | 7.96e-08 | 4.19e-07 | -0.437 | ko01100//Metabolic pathways;ko01240//Biosynthesis… |
| Liver | 14 dpi vs 7 dpi | Oleic acid | 12.1 | 2.70e-07 | 1.25e-06 | -0.679 | ko01060//Biosynthesis of plant secondary metaboli… |
| Liver | 14 dpi vs 7 dpi | L-Carnitine | 10.9 | 3.16e-12 | 6.59e-11 | -0.371 | ko04976//Bile secretion;ko04714//Thermogenesis;ko… |
| Liver | 14 dpi vs 7 dpi | Acetyl-DL-carnitine | 10.7 | 1.75e-13 | 6.11e-12 | -0.423 | ko04931//Insulin resistance |
| Liver | 14 dpi vs Control | Taurocholate | 17.8 | 3.16e-11 | 3.42e-10 | -0.292 | ko01100//Metabolic pathways;ko04976//Bile secreti… |
| Liver | 14 dpi vs Control | Hypoxanthine | 14.2 | 6.15e-17 | 8.24e-15 | 1.668 | ko01100//Metabolic pathways;ko01232//Nucleotide m… |
| Liver | 14 dpi vs Control | Dl-lactate | 12.2 | 1.18e-13 | 3.01e-12 | -1.442 | - |
| Liver | 14 dpi vs Control | Inosine | 12.1 | 1.41e-07 | 5.97e-07 | -1.184 | ko01100//Metabolic pathways;ko02010//ABC transpor… |
| Liver | 14 dpi vs Control | Acetyl-DL-carnitine | 11.9 | 6.14e-18 | 1.78e-15 | 1.030 | ko04931//Insulin resistance |
| Liver | 14 dpi vs Control | L-Carnitine | 11.2 | 1.19e-13 | 3.02e-12 | 0.669 | ko04976//Bile secretion;ko04714//Thermogenesis;ko… |
| Liver | 7 dpi vs Control | Taurocholate | 35.0 | 5.75e-17 | 1.17e-14 | -1.068 | ko01100//Metabolic pathways;ko04976//Bile secreti… |
| Liver | 7 dpi vs Control | Acetyl-DL-carnitine | 17.8 | 4.61e-18 | 2.59e-15 | 1.453 | ko04931//Insulin resistance |
| Liver | 7 dpi vs Control | L-Carnitine | 17.4 | 2.04e-17 | 6.02e-15 | 1.039 | ko04976//Bile secretion;ko04714//Thermogenesis;ko… |
| Liver | 7 dpi vs Control | Hypoxanthine | 15.5 | 2.18e-14 | 1.10e-12 | 1.557 | ko01100//Metabolic pathways;ko01232//Nucleotide m… |
| Liver | 7 dpi vs Control | Dl-lactate | 12.4 | 9.58e-13 | 2.48e-11 | -0.976 | - |
| Liver | 7 dpi vs Control | Niacinamide | 11.2 | 4.94e-07 | 2.32e-06 | 0.322 | ko01100//Metabolic pathways;ko01240//Biosynthesis… |
| Liver | 7 dpi vs Control | Inosine | 10.3 | 7.21e-13 | 1.94e-11 | 0.376 | ko01100//Metabolic pathways;ko02010//ABC transport… |

Note. Metabolites were selected from high-VIP annotated features and pathways relevant to bile acid, lipid, amino-acid, energy, and purine/nicotinamide metabolism. FDR values are provided to indicate the robustness of individual metabolite changes after multiple-testing correction.

| **Supplementary Table S6. Agreement between individual-fish and tank-level metabolomic effect estimates.** | | | | | | | | |
| --- | --- | --- | --- | --- | --- | --- | --- | --- |
| **Tissue** | **Contrast** | **Number of matched features** | **Spearman rho** | ***P* value** | **Original differential metabolites** | **Matched original differential metabolites** | **Concordant metabolites** | **Directional concordance** |
| Liver | 7 dpi vs Control | 19923 | 0.975828436 | < 2.2e-16 | 673 | 673 | 673 | 1 |
| Liver | 14 dpi vs Control | 19923 | 0.981130849 | < 2.2e-16 | 798 | 798 | 798 | 1 |
| Liver | 14 dpi vs 7 dpi | 19923 | 0.977917315 | < 2.2e-16 | 783 | 783 | 783 | 1 |
| Spiral valve intestine | 7 dpi vs Control | 22716 | 0.967216531 | < 2.2e-16 | 729 | 728 | 727 | 0.998626374 |
| Spiral valve intestine | 14 dpi vs Control | 22716 | 0.974550941 | < 2.2e-16 | 776 | 774 | 773 | 0.99870801 |
| Spiral valve intestine | 14 dpi vs 7 dpi | 22716 | 0.976065663 | < 2.2e-16 | 788 | 784 | 784 | 1 |

**Note:** Metabolite effect directions were highly consistent between the fish-level and tank-level analyses.
